# Abstracts of the Total Body PET conference 2018: Ghent, Belgium. 30 June - 2 July 2018

**DOI:** 10.1186/s40658-018-0218-7

**Published:** 2018-06-29

## Part 1: Invited Speakers

### S1 Total-Body Positron Emission Tomography: New Opportunities through Unprecedented Sensitivity

#### Simon R. Cherry^1,2^, Terry Jones^2^, Joel S. Karp^3^ and Ramsey D. Badawi^2,1^

##### ^1^Department of Biomedical Engineering, University of California, Davis, California, USA; ^2^Department of Radiology, University of California Davis Medical Center, Sacramento, California, USA; ^3^Department of Radiology, University of Pennsylvania, Philadelphia, Pennsylvania, USA

###### **Correspondence:** Simon R. Cherry

Positron emission tomography (PET) studies are largely limited by statistical noise caused by the relatively small number of recorded events, and the uncertainty in the location of these events due to limited timing resolution. Very large increases in detection sensitivity are possible by increasing the axial field of view from the 15-25 cm common today, to dimensions that cover the entire thorax or even the entire body. Further significant increases in effective sensitivity also are possible by increasing the time-of-flight resolution.

A number of academic groups and companies are now engaged in development of extended axial field of view and total-body PET systems. This presentation sets forth the case for total-body PET imaging, and examines a range of strategies that could lead to imaging of the entire human body with effective sensitivities up to two orders of magnitude higher than is possible with current generation PET/CT scanners. This step change in performance offers enormous opportunities for molecular imaging to extend its reach and impact in both clinical diagnostics and research.

### S2 The PennPET Explorer with Scalable Axial Field-of-View: Design and Initial Measurements

#### J. S. Karp^1^, V. Viswanath^1^, J.P. Schmall^1^, M. E. Werner^1^, M. J. Parma^1^, S. Matej^1^, M. E. Daube-Witherspoon^1^, T. McDermott^1^, M. J. Geagan^1^, G. Muehllehner^1^, C-H Tung^2^, A. E. Perkins^2^

##### ^1^Department of Radiology, University of Pennsylvania, Philadelphia, PA, USA; ^2^Philips Healthcare, Highland Heights, OH, USA

**Objectives:** The EXPLORER Consortium is developing two systems with very large axial fields -of-view (AFOV) to enhance performance for clinical applications, and to enable research applications that require simultaneous measurement of larger volumes and multiple organ systems within the body. The UC Davis Explorer will be capable of total-body imaging at high spatial resolution, whereas the system under development at U Penn will be capable of complete torso imaging at high TOF resolution. This study is to characterize the imaging performance of the PennPET Explorer in its current configuration with a 70 cm AFOV.

**Methods:** The PennPET Explorer is designed as a scalable, multi-ring system where each ring has 22.9 cm axial length, thus 3 rings results in 70 cm AFOV. The detector tiles consist of 8×8 arrays of 3.86×3.86× 19 mm^3^ LYSO crystals coupled in a 1-to-1 configuration to the Philips Digital Photon Counting (PDPC) SiPM device. The tiles are operated at a temperature of 5°C using chilled water for the detector plates and circulating dry air in the detector bays to avoid condensation on the electronics. The system acquires data as singles events from each ring, and rings are time synchronized to enable off-line sorting into coincidence pairs from all combinations of rings. We have adapted both our list-mode TOF OSEM reconstruction algorithm for the large AFOV data as well as the DIRECT histo -image based reconstruction approach, which includes a tilt-dependent axial resolution model to mitigate losses in spatial resolution due to axial parallax error.

**Results:** We have demonstrated a superb timing resolution of 250 ps, along with an energy resolution of 11% and a spatial resolution of 3.9 mm. We have performed multi -ring data acquisitions and reconstructed phantom images with all coincidence pairs to achieve excellent image quality with shorter scan times.

**Conclusions:** A large AFOV PET system has been designed, with consideration for developing a practical instrument that meets the requirements of the intended end -users. Initial measurements of the prototype scanner design indicate excellent performance and we have completed major milestones, specifically data acquisition and image reconstruction for the 70 cm AFOV configuration. The PET scanner will soon be integrated with a couch and CT to enable human imaging and initiation of clinical research studies in the near future. We plan to extend the AFOV beyond 70 cm at a later time to further extend the capabilities of the PennPET Explorer for both adult and pediatric body imaging.

**Support:** We acknowledge Philips Healthcare, NIH R01 CA206187 and NIH R01-CA113941.

### S3 The UC Davis EXPLORER Project

#### Simon R. Cherry^1,2^, Eric Berg^1^, Martin S. Judenhofer^1^, Xuezhu Zhang^1^, Julien Bec^1^, Jinyi Qi^1^, Terry Jones^2^ and Ramsey D. Badawi^2,1^

##### ^1^Department of Biomedical Engineering, University of California, Davis, California, USA; ^2^Department of Radiology, University of California Davis Medical Center, Sacramento, California, USA

###### **Correspondence:** Simon R. Cherry

The goal of the UC Davis EXPLORER project is to develop the world’s first total-body positron emission tomography (PET) scanner and to demonstrate the value of total-body PET imaging for both existing and new applications. The project has resulted in the construction of three scanners, two small-scale prototypes and the 2-meter long human EXPLORER scanner. A mock-up of the human scanner also has been developed to study issues related to patient comfort, claustrophobia, subject motion, workflow, and logistics of tracer injection and blood sampling.

The first prototype, miniEXPLORER I, was developed in collaboration with Siemens and utilizes detectors and electronics from the mCT PET/CT scanner, reconfigured into a system with 43.5 cm diameter detector rings and an axial length of 45.7cm. The measured performance at the system level showed an energy resolution of 12%, timing resolution of ~600 psecs and a spatial resolution of 3.0 mm. The peak sensitivity at the center of the scanner was 15% and the sensitivity using the NEMA NU 2-2012 standard was 50 kcps/MBq. This system is installed at the California National Primate Research Center, where it has been used for dynamic whole-body imaging studies in nonhuman primates with a range of radiotacers.

The second prototype, miniEXPLORER II, was developed with United Imaging Healthcare, and was the test platform for all the components of the human EXPLORER scanner. This system has a ring diameter of 52 cm, an axial field of view of 48.3 cm and is installed at the School of Veterinary Medicine at UC Davis. The performance evaluation demonstrated energy resolution of 11.7%, timing resolution of 409 psec and a spatial resolution of 2.6 mm. The peak sensitivity at the center of the scanner is 16% and the NEMA NU2-2012 sensitivity was 55 kcps/MBq. The first companion animal study has been completed.

The human EXPLORER scanner is currently under construction. It will consist of 564,480 LYSO detector elements each measuring 2.76 x 2.76 x 18.1 mm, read out by 53,760 silicon photomultipliers. The ring diameter is 78.6 cm (patient bore of 70 cm) with an axial length of 195 cm. The system also includes a 128-slice CT scanner for anatomic correlation and attenuation correction.

Finally, a range of possible clinical and research applications for the EXPLORER scanner has been identified. Some of these are currently being prototyped using the miniEXPLORER scanners, while others await the completion of EXPLORER which is expected mid 2018.

### S4 Monolithic high-resolution total-body PET

#### Stefaan Vandenberghe

##### MEDISIP, Ghent University, Ghent, Belgium

**Background**

Nearly all clinical whole body and total body PET systems are based on pixelated detector technology. Monolithic detectors have been introduced in small animal imaging systems and the potential of this detector technology with regards to excellent spatial resolution has been illustrated in several commercial systems. This technology has also large potential for clinical systems as it can combine high spatial resolution, depth-of-interaction information and good TOF performance.

**Materials and methods**

The final spatial resolution of a PET system is determined by a combination of physics effects (positron range and acolinearity) in convolution with detector performance. To reach the limits for a clinical system (just below 2 mm) a detector with an intrinsic spatial resolution of 1.3-1.5 mm is ideal. The current monolithic detector (25 mm x 25 mm x 8 mm thick) has a resolution well below 1mm, but has limited stopping power due to its limited thickness. Therefore the monolithic detector the aspect ratio is kept similar and the detector is increased by a factor 2 in both transverse and axial dimensions. By repeating this block 36 times in one ring, a system with 65 cm inner diameter is obtained. Based on this detector a total body PET system was designed and simulated using GATE.

**Results and Conclusions**

Several sources were simulated to determine the system performance for imaging compact and extended sources. The system requires 3-4 more scintillation and readout than current clinical PET systems but outperforms it with regards to sensitivity (and spatial resolution). Gains comparable to the extension in axial length are obtained for compact sources. Large increases (15-20x) are seen for extended sources.

### S5 J-PET: Towards total-body modular PET from plastic scintillators

#### Pawel Moskal

##### Faculty of Physics, Astronomy and Applied Computer Science, Jagiellonian University, S. Łojasiewicza 11, 30-348, Kraków, Poland

**Background**

Positron emission tomography (PET) is a well established medical diagnostics method. It is, however, very expensive [1], in part due to the very high costs of the currently used commercial PET scanners which all are based on the relatively expensive inorganic crystals [2-4]. The high cost of PET is also one of the barriers for the use of this modality with the large axial field-of-view which would enable single-bed imaging of the whole human body. At present there is an ongoing research conducted in the frame of the EXPLORER project aiming at building the first total body PET based on crystal detectors [5-7].

In this talk a proposition of the usage of plastic scintillators as a detection material for the positron emission tomography will be presented [8-14]. Tomograph built from axially arranged plastic scintillator strips may allow for the construction of a cost effective total-body scanner due to the less expensive detector material, and reduced number of the electronic channels.

**Materials and methods**

Jagiellonian Positron Emission Tomograph (J-PET) is the first PET built from plastic scintillators [8-14]. The J-PET prototype consists of 192 detection modules arranged axially in three layers forming a cylindrical diagnostic chamber with the inner diameter of 85 cm and the axial field-of-view of 50 cm. Axial arrangement of long strips of plastic scintillators, their small light attenuation, superior timing properties, and relative ease of increasing the axial field-of-view opens promising perspectives for a cost effective construction of the total-body PET scanner, as well as construction of MR and CT compatible PET inserts.

**Results and Conclusions**

Status of the commissioning of the first J-PET prototype as well as status of the development of the second fully modular and portable J-PET tomograph will be presented and discussed. The modularity and light weight of the J-PET shall enable adjustment of the size of the diagnostic chamber to the size of the patient.

In the talk we will present the method of photon registration, fully digital signal processing and data acquisition, as well as methods of event selection and image reconstruction. Additionally, we will argue that J-PET shall enable imaging of positronium properties based on three-photon e+e- annihilations [15]. We will present results [16-19] of feasibility studies of positronium imaging, showing that it may deliver new diagnostic informations, additonal to the presently available standardised uptake value indicator.

**Acknowledgements**

The results presented here are on behalf of the J-PET collaboration.

We acknowledge the financial support by The Polish National Center for

Research and Development through grant INNOTECH-K1/IN1/64/159174/NCBR/12, the Foundation for Polish Science through the MPD and TEAM programmes, the National Science Centre through grants Nos. 2016/21/B/ST2/01222, 2017/25/N/NZ1/00861, and the Ministry for Science and Higher Education through grants No. 6673/IA/SP/2016, 7150/E-338/SPUB/2017/1.

**References**

[1] A. K. Buck et al., J. Nucl. Med. **51**, 401 (2010).

[2] J. S. Karp et al., J. Nucl. Med. **49**, 462 (2008).

[3] P. J. Slomka et al., Semin. Nucl. Med. **46**, 5 (2016).

[4] S. Vandenberghe et al., EJNMMI Phys. **3**, 3 (2016).

[5] S. R. Cherry et al., Sci. Trans. Med. **9**, eaaf6169 (2017).

[6] X. Zhang et al., Phys. Med. Biol. **62**, 2465 (2017).

[7] V. Viswanath et al., Acta Phys. Polon. **B 48**, 1555 (2017).

[8] P. Moskal et al., Patents: US8969817; US8859973; US9804206; US9804279; US9804274; US9798021;

[9] J-PET: P. Moskal et al., Nucl. Instrum. Meth. **A 764**, 317 (2014)

[10] J-PET: P. Moskal et al., Nucl. Instrum. Meth. **A 775**, 54 (2015)

[11] J-PET: P. Moskal et al., Phys. Med. Biol. **61**, 2025 (2016)

[12] J-PET: J. Smyrski et al., Nucl. Instrum. Meth. ***A*** **851**, 39 (2017)

[13] J-PET: L. Raczyński et al., Phys. Med. Biol. **62**, 5076 (2017)

[14] J-PET: Sz. Niedzwiecki et al., Acta Phys. Polon. **B 48**, 1567 (2017)

[15] P. Moskal et al., Patents: US9851456, PL 227658

[16] J-PET: A. Gajos et al., Nucl. Instrum. Meth. ***A*** **819**, 54 (2016)

[17] J-PET: D. Kamińska et al., Eur. Phys. J*.* **C 76**, 445 (2016)

[18] J-PET: B. Jasinska et al., Acta Phys. Polon. **B 48**, 1731 (2017)

[19] B. Jasinska, P. Moskal, Acta Phys. Polon. **B 48**, 1577 (2017)

## Part 2: Regular abstracts

### A1 Single-chip tomographic data processing platform

#### Grzegorz Korcyl (grzegorz.korcyl@uj.edu.pl)

##### Department of Information Technologies, Jagiellonian University, Kraków, Poland

**Background**

Efforts in tomographic data processing, both scientific and commercial are directed towards fastest generation of high-quality images. For this purpose, many sophisticated algorithms have been developed with Maximum Likelihood Expectation Maximization (MLEM) and Ordered Subsets MLEM (OSEM) being mostly evaluated [1, 2]. They are heavy computational iterative procedures, therefore requiring significant CPU and GPU power in order to deliver reconstructed image within reasonable amount of time [3].

Current developments in scanners technology, mostly the researches on whole-body and three-dimensional data acquisition create new challenges for data processing systems. Extended Field-Of-View (FOV) and voxelization of large volumes renders current techniques inefficient in terms of required computing power and memory capacity and consequently required space, increased power consumption and costs [4, 5].

**Materials and Methods**

Presented project is a new approach to the tomographic data processing chain in which all necessary steps towards image reconstruction are enclosed in a compact package working in real-time regime.

The introduction of System-on-Chip FPGA devices with integrated ARM processors [6], allows to benefit from high amount of programmable logic resources for real-time 2.1.1.2.processing of the raw detector data and run high-level data analysis in the same time and in the same chip.

**Results**

We have developed a proof-of-concept system and demonstrated its operation on the J-PET scanner [7, 8]. The system processes 8 data streams performing following steps:

- Parsing raw data from digitizing electronics
- Extraction of the hits on the scanner channels
- Application of scanner geometry and calibration parameters
- Coincidence search for Line-of-Response (LOR) candidates
- Region-of-Response (ROR) calculation
- Transfer ROR data to the shared memory for visualization
- Transfer ROR data to external storage
- Visualization of the gathered data in a form of histograms and 3D point cloud

The measurements show that the system is capable to process up to 42 MHits per second. Comparison between GATE simulations and measurements show agreement in terms of the estimated registered LORs and the number of LORs processed in a measurement under the same conditions. Data quality is verified by comparing a naïve reconstruction performed in the FPGA logic to the result of MLEM, computed on the same data set.

**Conclusions**

In the presence of whole-body, three-dimensional scanners, there is a need for exploring alternative data processing solutions. Computing platforms based on FPGA devices are perfectly suitable for processing multiple data streams in real-time, significantly reduce the generated data volume and generate instant visualization of the measurement.

**Acknowledgments**

This project has been developed on behalf of the J-PET Collaboration.

TRB platform together with accompanying firmware and software is developed by the TRB3 Collaboration (trb.gsi.de).

This project could be realized thanks to the support from Xilinx University Program and Altera University Program.

We acknowledge support by the National Science Centre through the grant No. 2016/21/B/ST2/01222, by the National Centre for Research and Development through grants Nos. INNOTECH-K1/IN1/64/159174/NCBR/12, LIDER/274/L-6/14/NCBR/2015 and by the Ministry for Science and Higher Education through grants Nos. 6673/IA/SP/2016 - IA/SP/01555/2016, 7150/E-338/SPUB/2017/I and The Foundation for Polish Science (MPD).

**References**

1. L. A. Shepp, Y. Vardi, “Maximum Likelihood Reconstruction for Emission Tomography”, *Trans. Med Imaging*, vol. 1.2, pp. 113-122, Oct. 1982

2. H. M. Hudson, R. S. Larkin, “Accelerated image reconstruction using ordered subsets of projection data”, *IEEE Trans. Med. Imaging*, vol. 13, pp. 601-609, 1994

3. B. Goldschmidt, *et al.*, “Software-Based Real-Time Acquisition and Processing of PET Detector Raw Data”, *IEEE Trans Biomedical Eng*., vol. 63, pp. 316- 326, Feb. 2016

4. P. Slomka, T. Pan, G. Germano, “Recent Advances and Future Progress in PET Instrumentation”, *Semin. Nucl. Med.*, vol. 46, 2016

5. S. R. Cherry, *et al.*, “Total-Body PET: Maximizing Sensitivity to Create New Opportunities for clinical Research and Patient Care”, *J. Nucl. Med.,* vol. 59, pp. 3-12, Jan, 2018

6. B. Dammak, *et al.*, “Hardware Resource Utilization Optimization in FPGA Heterogeneous MPSoC Architectures”, *Microprocessors and Microsystems*, vol. 39, pp. 108-1118, June 2015

7. P. Moskal, *et al.*, “Novel detector systems for the Positron Emission Tomography”, *Bio-Algorithms and Med-Systems*, vol. 7, pp. 73-78, 2011

8. Sz. Niedzwiecki, *et al.*, “J-PET: A New Technology for the Whole-Body PET Imaging”, *Acta Phys. Polon. B.*, vol. 48, pp. 1567-1576, 2017

### A2 Timing calibration in TOF-PET using data consistency: the 3D case

#### Michel Defrise^1^, Ahmadreza Rezaei^2^, Johan Nuyts^2^

##### ^1^Department of Nuclear Medicine, Vrije Universiteit Brussel, B-1090, Brussels, Belgium; ^2^Department of Nuclear Medicine, KU Leuven, B-3000, Leuven, Belgium

###### **Correspondence**: Michel Defrise

The time calibration of a TOF-PET scanner is usually done by measuring a known activity distribution [1, 2, 3, 4]. Alternative, data driven, methods can be useful to monitor possible drifts of the TOF offsets directly from clinical data. A simple data driven method [5], [6] is the indirect method, which exploits the fact that the TOF summed data are not affected by a timing bias. Recently [7] we introduced a faster direct data driven calibration method for a single ring PET scanner, which does not require a non-TOF reconstruction. The algorithm was derived for a continuous 2D model, assuming as in [1, 2, 3, 4, 5] that the TOF misalignment of each LOR is the difference between the timing offsets of the two crystals in coincidence. The consistency equations for TOF PET [8] lead to a relation between the data and the crystal offsets, which is linear, only involves the two first moments of the TOF data, and is independent of the TOF resolution. Although derived for a continuous model the method was successfully implemented for a simulated single ring scanner with 48 blocks of 13 detectors, separated by a gap of one detector. We will present numerical results from [7], which show that this consistency based calibration, while not matching the accuracy of the indirect method, nevertheless recovers the timing offsets with small errors compared to the TOF resolution and have a limited impact on OSEM reconstructions.

The present work generalizes the method to an arbitrary 3D scanner geometry, assuming as in 2D that the detector sampling is sufficiently fine. We prove the following property. Denote the fully corrected data for the LOR linking two detectors located at $\overrightarrow{a}\in {R}^3$and $\overrightarrow{b}\in {R}^3$as

1 $$ m\left(\overrightarrow{a},\overrightarrow{b},t\right)=\int dl\ w\left(t-\eta \left(\overrightarrow{a}\right)+\eta \left(\overrightarrow{b}\right)-l\right)\ f\left(\overrightarrow{a}+\frac{\left(l+L/2\right)\left(\overrightarrow{b}-\overrightarrow{a}\right)}{L}\right) $$

with *f* the activity, $L=\left\Vert \overrightarrow{b}-\overrightarrow{a}\right\Vert$ and $\eta \left(\overrightarrow{a}\right),\eta \left(\overrightarrow{b}\right)$the offsets of the two detectors, and we assume an even TOF profile *w(t) = w(-t)*. Define

$$ {M}_0\left(\overrightarrow{a},\overrightarrow{b}\right)=\int dt\ m\left(\overrightarrow{a},\overrightarrow{b},t\right)\kern0.5em ,\kern0.5em {M}_1\left(\overrightarrow{a},\overrightarrow{b}\right)=\int dt\ t\ m\left(\overrightarrow{a},\overrightarrow{b},t\right) $$

the two moments of the data. Then the offsets *η* must be solution of the following equation (*∇*_*a*_denotes the gradient with respect to the detector location $\overrightarrow{a}$):

2 $$ \frac{2}{L}\left({\nabla}_a+{\nabla}_b\right)\left({M}_1-\left(\eta \left(\overrightarrow{a}\right)-\eta \left(\overrightarrow{b}\right)\right){M}_0\right)+L\left({\nabla}_a-{\nabla}_b\right)\frac{M_0}{L} $$

This generic equation can be specialized for any 3D scanner geometry (the case of a cylindrical scanner will be presented), and discretized as a system of N_detector_ linear equations for the N_detector_ offsets **η**. Implementation issues will be discussed, in particular:

- Integrating (2) over $\overrightarrow{b}$at fixed $\overrightarrow{a}$ to obtain a well conditioned integral equation (as in [7] in 2D),
- The segmentation of this system of equation for scanners with very large axial FOV.

**References**

[1] Perkins A E,Werner M, Kuhn A, Surti S, Muehllehner G and Karp J S. Time-of-flight coincidence timing calibration techniques using radioactive sources. IEEE Nucl. Sci. Symp. Conf. Rec. 2005; 5: 2488-91

[2] Thompson C J, Camborde M and Casey M E. A central positron source to perform the timing alignment of detectors in a PET scanner. IEEE Trans. Nucl. Sci. 2005; 52: 1300-4.

[3] Li X, Burr K C, Wang G-C, Du H, Gagnon D. Timing Calibration for Time-of-Flight PET Using

Positron-Emitting Isotopes and Annihilation Targets. IEEE Trans. Nucl. Sci. 2016; 63:1351-1358.

[4] Yu X, Isobe T, Watanabe M and Liu H. Novel crystal timing calibration method based on total variation. Phys. Med. Biol. 2016; 61: 7833-7847.

[5] Werner M E and Karp J S. TOF PET offset calibration from clinical data. Phys. Med. Biol. 2013; 58: 4031-46.

[6] Rezaei A, Schramm G and Nuyts J. Data driven time alignment for TOF-PET, Records of the 2017 IEEE Medical Imaging Conference, Atlanta (GA).

[7] Defrise M, Rezaei A, Nuyts J. Time-of-flight PET time calibration using data consistency. To appear in Phys Med Biol 2018.

[8] Defrise M, Panin V Y and Casey M E. New Consistency Equation for Time-of-Flight PET. IEEE Trans. Nucl. Sc 2013; 60: 124-33.

### A3 Pilot evaluation of the MINDView brain PET insert, based on monolithic LYSO blocks, in a 3T MRI

#### A. J. Gonzalez^1^, A. Gonzalez-Montoro^1^, J. Barbera^2^, L. Moliner^1^, L. F. Vidal^1^, E. J. Pincay^1^, G. Cañizares^1^, E. Lamprou^1^, S. Sanchez^1^, F. Sanchez^1^, C. Correcher^3^, J. V. Catret^2^, S. Jimenez^2^, S. Aussenhofer^4^, J. Cabello^5^, M. Schwaiger^5^, A. Iborra^6^, J. M. Benlloch^1^

##### ^1^Instituto de Instrumentación para Imagen Molecular (I3M), CSIC — Universitat Politècnica de València, 46022, Valencia, Spain; ^2^Oncovision, 46022, Valencia, Spain; ^3^Bruker Biospin, 46013, Valencia, Spain; ^4^NORAS MRI Products GmbH, Hochberg, Germany; ^5^Nuklearmedizin, Klinikum rechts de Isar, Technische Universität München, Munich, Germany; ^6^Brest INSERM, UMR1101, LaTIM, Université de Bretagne Occidentale, Brest, France

**Background**

We report in this work the current status performance of the brain PET insert developed under the MINDView project. Final construction has been accomplished. This PET insert uses, to our knowledge, the highest number of monolithic blocks in a scanner, a total of 60, with total LYSO volume of 3,000 cm^3^ and 1,440 signals.

**Materials and Methods**

Pilot performance studies have been carried out at the lab, partially following the NEMA protocol. It has later been installed at the nuclear medicine department in Klinikum rechts der Isar (Munich) and exhaustively tested inside the Siemens mMR, a whole body PET-MR with a 3T main magnetic field. The PET insert FOV is 154 mm axially and 240 mm transaxially, defined by 3 rings of 20 monolithic crystals each (50 x 50 x 20 mm), coupled to custom 12x12 SiPM arrays, and readout through custom electronics providing information of two projections of the scintillation light (X and Y).

**Results**

The system sensitivity is above 7% (350-650 keV) with a point-like source at the CFOV, and increases to about 10% for the range of (250-750 keV). Average energy resolution of the entire scintillation volume is about 17%. The spatial resolution, measured with the 0.25 mm NEMA source across the radial direction, showed values below 2 mm.

A variety of MR sequences for brain imaging have been run (EPI, ASL, T1w, T2w and UTE), and the PET response measured, without showing any deterioration. Count rates as a function of sequences were studied not also exhibiting a system deterioration. Also the MR performance has been studied, among other tests the uniformity of the B0 and B1 fields, not showing significant changes when the dedicated brain PET is inserted.

Regarding performance comparison with a state-of-the-art whole body PET system, it shows an improved performance as observed through the mini-Derenzo or other phantoms. Rods of about 2 mm are clearly distinguished with standard iterative reconstruction methods and voxel/pixel sizes.

**Conclusions**

We have designed and implemented a brain PET insert using 60 large monolithic LYSO blocks, defining a large FOV with uniform performance in it, and not showing any deterioration when brain imaging MR sequences are used, including EPI or UTE. Detailed analyses will be presented. Currently, the system is in Klinikum rechts der Isar (Munich) and patients recruitment is undergoing.

### A4 Full body intelligent scanning preclinical PET: characterization and first animal tests of a small-scale system

#### P. M. M. Correia^1^, F. M. Ribeiro^1^, J. Menoita^1^, A. L. M. Silva^1^, N. O. Romanyshyn^1^, F. Rolo^1^, I. F. Castro^2^, P. M. C. C. Encarnação^2^, F. Rodrigues^2^, A. C. Santos^3^, C. Ramos^3^, F. Caramelo^3^, N. C. Ferreira^3,4^, D. A. Sá^3,4^, N. Matela^5^, P. Almeida^5^, P. M. Sá^5^, J. F. C. A. Veloso^1^

##### ^1^i3N – Departamento de Física da Universidade de Aveiro, 3810-193, Aveiro, Portugal; ^2^RI-TE - Radiation Imaging Technologies, Lda, UA Incubator, PCI – Creative Science Park, 3830-352, Ílhavo, Portugal; ^3^IBILI/FMUC - Instituto de Biofisica/Biomatemática, Faculdade de Medicina da Univ. de Coimbra 3000-545, Coimbra, Portugal; ^4^ICNAS, Universidade de Coimbra 3000-545, Coimbra, Portugal; ^5^Instituto de Biofísica e Engenharia Biomédica, Faculdade de Ciências, Universidade de Lisboa, 1749-016, Lisboa, Portugal

###### **Correspondence:** J. F. C. A. Veloso (joao.veloso@ua.pt)

**Background**

A new concept of preclinical PET scanner using an innovative acquisition method based on two rotation axes for the movement of detector pairs is being developed for whole body small animal imaging purposes. An intelligent scanning can be applied as a function of the imaged subject. This innovative concept allows achieving high and uniform position resolution over the whole field of view (FoV), by eliminating parallax errors due to the depth of interaction, which are typical of ring-based PET systems. The absence of parallax effect in transaxial plane does not impose limitations on the proximity of the detector elements to the FoV favoring the system sensitivity. Full axial imaging is possible using only a small number of detector elements, allowing for an unprecedented performance/cost ratio. A demonstrator prototype was built with 16 + 16 detector cells, based on LYSO scintillators coupled to SiPMs, covering a FoV of 50 mm Ø × 35 mm. Patent: PCT/IB2016/051487

**Materials and Methods**

Detector blocks are made of linear arrays of 16 LYSO with 2×2×30 mm^3^ coupled to individual SiPMs, with custom-developed readout electronics. Spatial resolution (radial and tangential) was measured according to NEMA NU4-2008 standards, using a 0.25 mm Ø ^22^Na source embedded in a 1 cm^3^ PMMA and with a 25 μCi activity. A Micro-PET Image Quality (IQ) phantom with 400 μCi of FDG was imaged following the NEMA NU4-2008. The acquisition was performed covering the entire phantom. Filtered Back Projection (FBP) reconstruction method was used for the spatial resolution and IQ phantom acquisitions. Animal imaging tests were carried out on a 27 g mouse injected with 200 μCi of FDG.

**Results**

The spatial resolution obtained in radial and tangential directions was around 1 mm over the entire FoV. This result compares favorably with ring-based micro-PET scanners using 2×2 mm^2^ scintillators. The IQ phantom reveals details that allow us to identify the 1 mm Ø filled FDG rod, demonstrating the excellent image quality of the system, even considering the small number of detector cells used. Mouse imaging reveals details that show the high image quality and useful information that can be extracted from the system, Fig. 1.

**Conclusion**

The results indicate that a spatial resolution below 1 mm can be reached for the entire FoV (using FBP reconstruction) as well as good quality details observed in animal imaging, indicating very promising prospects for the development of a high performance preclinical system for small animal imaging.

**Keywords**

PET; SIPM; DOI; easyPET; preclinical

**Acknowledgements**

This work was partially supported by projects POCI-01-0145-FEDER-016855 and PTDC/BBB-IMG/4909/2014, CENTRO-01-0247-FEDER-017823, and CENTRO-01-0145-FEDER-000003, through CENTRO2020, COMPETE, FEDER, POCI and FCT (Lisbon) programs.

**Fig. 1 (abstract A4).**
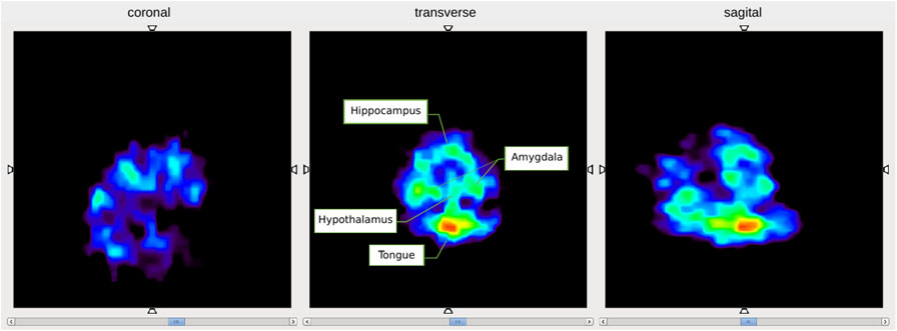
Example of a brain image of the 27 g mouse

### A5 Time Over Thresholds as a measure of energy loss by incident gamma in the J-PET scanner

#### Sushil K. Sharma^1^, Sz. Nied*ź*wiecki^1^

##### ^1^M. Smoluchowski Institute of Physics, Jagiellonian University, Lojasiewicza 11, 30-348 Cracow, Poland

###### **Correspondence:** Sushil K. Sharma (sushil.sharma@uj.edu.pl)

**Background**

The Jagiellonian-Positron Emission Tomograph (J-PET) is the first PET composed of plastic scintillators. J-PET consists of 192 individual modules of dimension 500 X 19 X 7 mm^3^. The modules are arranged axially in the three layers of diameter 85, 93.5 and 115 cm respectively. The long strips of plastic scintillators used in J-PET provide superior timing properties, small light attenuation, larger axial field-of-view (AFOV) and thus qualify for the use in whole-body PET Imaging [1, 2, 3]. Moreover, using plastics as detecting material allow for constructing a cost-effective whole body scanner. A major advantage of the plastic scintillator is that the signals are very fast [4]. Such signals allow for superior time resolution and decrease the pile-ups with respect to crystal-based detectors. In order to take advantage of excellent timing properties of plastic scintillators, in the frame of J-PET the charge collection is replaced with time over threshold (TOT) measurements, which is a well-established method for the signal processing particularly in multi-channel readout systems [5,6].

**Materials and methods**

The challenge in adopting the TOT technique with plastic scintillators is due to the partial deposition of energy by incident gamma as they interact predominantly via Compton scattering. However, implementing the Multi-Voltage-Threshold (MVT) and probing the signal at four different thresholds help greatly in improving the energy loss resolution [5] of tomograph. In order to determine the relationship between TOT and energy loss, the data was collected using ^22^Na source placed at the center of tomograph surrounded by porous material. The geometrical acceptance of J-PET offers the possibility to study the scattering of incident gamma.

**Results**

The algorithm used for the analysis allows to tag the energy and scattering angle of the incident gamma which in turn gives the estimate about the energy deposition in the scintillator. Thus for each interaction in the scintillator one can obtain one-to-one information of the energy deposition by the gamma photon and corresponding TOT values.

**Conclusions**

We have established the relationship between TOT and energy loss by gamma quanta in the J-PET scanner built from the plastic scintillators. The relationship obtained from the analysis of experimental data can be well described by using the function TOT = A + B * ln(E_loss_).

**Acknowledgement**

The results presented here are on the behalf of J-PET collaboration. We acknowledge the support of the National Science Centre through the grant No. 2016/21/B/ST2/01222, by the National Centre for Research and Development through grants Nos. INNOTECH-K1/IN1/64/159174/NCBR/12, by the Ministry for Science and Higher Education through grants Nos. 6673/IA/SP/2016 - IA/SP/01555/2016, 7150/E-338/SPUB/2017/I and The Foundation for Polish Science (MPD & TEAM).

**References**

[1] S. Niedzwiecki et al., Acta Physica Pol B 48, (2017) 1567 - 1576

[2] P. Moskal et al., Phys. Med. Biol. 61, (2016) 2025 - 2047

[3] P. Moskal et al., Bio-Alg. Med.-Sys. 7, (2011) 73 - 78

[4] A. Wieczorek et al., PLOS ONE 12(11): (2017) e0186728

[5] M. Palka et al., JINST 12, (2017) P08001

[6] G. Korcyl et al., Acta Phys. Polon. B 47, (2016) 491 - 496

### A6 Image reconstruction of the simulated NEMA IEC phantom in J-PET scanner using multivariate kernel density estimation

#### R. Y. Shopa (Roman.Shopa@ncbj.gov.pl)

##### Świerk Computing Centre, National Centre for Nuclear Research, Otwock-Świerk, Poland

Jagiellonian PET (J-PET) scanner is a novel PET detector with the large axial field of view, which operating principle is based on the Compton scattering of photons inside plastic scintillator strips [1,2]. The tomograph exhibits excellent time resolution below 100 ps and is expected to provide a superior figure of merit for the total body imaging. Current prototype comprises three cylindrical layers, each composed of 50-cm long scintillator strips. The ongoing work is to add another module with more complex geometry.

Software products for image reconstruction, developed mainly for commercial PET scanners, do not support long and continuous scintillator strips in J-PET, so that additional errors would be imposed. Besides, some reconstruction frameworks, such as STIR [3], do not incorporate time of flight (TOF), which is one of the advantages of J-PET. As a reasonable alternative, we utilize a statistical tool – kernel density estimation (KDE), for the points of positron and electron annihilation, estimated from TOF.

Figure 1 shows the transverse (XY) and the coronal (XZ) cross-sections of the NEMA IEC phantom [4], simulated inside the ideal 1-layer J-PET scanner in the GATE framework [5] and reconstructed using multivariate KDE from the “ks” package, developed for R software environment [6]. Spatial and temporal components of the data were smeared according to the perspective readout of the silicon photomultiplier matrices (SiPM), combined with wavelength shifters (WLS) [7].

Reconstructed 3D images for the IEC phantom, obtained for various readouts, were compared for both KDE and STIR algorithms. The advantages of TOF incorporation are clearly visible, since it produces satisfactory results even without filtering the data – by the exclusion of scattered and accidental events along with weighting added due to attenuation effects.

**Acknowledgements**

The results are presented on behalf of J-PET Collaboration [http://koza.if.uj.edu.pl/pet/].

**References**

1. Moskal P, Zoń N, Bednarski T, Białas P, Czerwińskia E, Gajos A et al. A novel method for the line-of-response and time-of-flight reconstruction in TOF-PET detectors based on a library of synchronized model signals. Nucl. Instr. Meth. Phys. Res. A. 2015 Mar 1; 775:54-62.

2. Moskal P, Rundel O, Alfs D, Bednarski T, Białas P et al. Time resolution of the plastic scintillator strips with matrix photomultiplier readout for J-PET tomograph. Phys. Med. Biol. 2016 Feb 19; 61(5):2025–2047.

3. Thielemans K, Tsoumpas C, Mustafovic S, Beisel T, Aguiar P, Dikaios N et al. STIR: software for tomographic image reconstruction release 2. Phys. Med. Biol. 2012 Feb 21; 57(4):867-883.

4. International Standard: Radionuclide imaging devices – Characteristics and test conditions – Part 1: Positron emission tomographs, International Electrotechnical Commission (IEC), 61675-1. Geneva, Switzerland; 1998.

5. Jan S, Santin G, Strul D, Staelens S, Assié K, Autret D et al. GATE: a simulation toolkit for PET and SPECT. Phys. Med. Biol. 2004 Oct 7; 49(19):4543-4561.

6. Duong T. ks: Kernel Smoothing [Internet]. 2018 Jan 16 [cited 2018 March 26]; Available from: http://cran.r-project.org/web/packages/ks/.

7. Smyrski J, Alfs D, Bednarski T, Białas P, Czerwiński E, Dulski K et al. Measurement of gamma quantum interaction point in plastic scintillator with WLS strips, Nucl. Instrum. Methods Phys. Res. A. 2017 Jan 24; 851:39-42.

**Fig. 1 (abstract A6).**
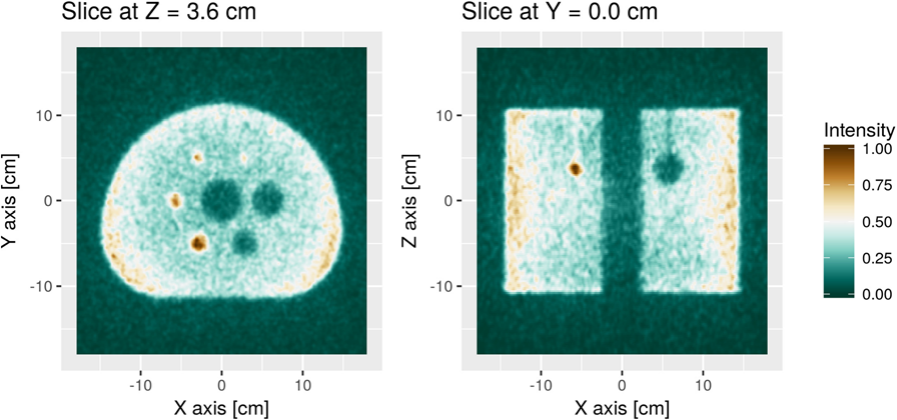
Transverse (left) and coronal (right) cross-sections of the reconstructed IEC phantom, simulated for the ideal J-PET scanner with SiPM+WLS readout, using multivariate KDE

### A7 Total body dynamic FDG PET imaging of spontaneously hypertensive rats

#### Qiao Huang^1^, Jie Li^1^, R. Jack Roy^1^, Mahendra D. Chordia^1^, Stan Majewski^1^, Stuart S. Berr^1,2^, Katelyenn McCauley^1^, Kiel Neumann^1^, Susanna R. Keller^3^, Bijoy K. Kundu^1,2^

##### ^1^Department of Radiology and Medical Imaging, University of Virginia, Charlottesville, VA 22908, USA; ^2^Department of Biomedical Engineering, University of Virginia, Charlottesville, VA 22908, USA; ^3^Department of Medicine-Endocrinology and Metabolism, University of Virginia, Charlottesville, VA 22908, USA

###### **Correspondence:** Bijoy K. Kundu (bkk5a@virginia.edu)

**Background:** Left ventricular hypertrophy (LVH) due to hypertension (HTN) is a key risk factor for the development of heart failure (HF). HF in turn can lead to decreased perfusion and consequently functional impairments of different tissues. In this study we evaluated myocardial and cerebral glucose metabolism, using dynamic FDG PET imaging, in the spontaneously hypertensive rat (SHR) at ~18 months of age. LV function in SHR exhibits compensation until ~17 months of age and systolic HF at ~20 months of age [1].

**Materials and Methods:** Dynamic FDG PET imaging [2] (~250-300 μCi) of rats at ~18 months of age was performed using the Bruker Albira Si Trimodal imager (150 mm axial FOV) [3]. The 60 minute list-mode data was histogrammed into 23 time bins and reconstructed with attenuation correction using MLEM algorithm. Regions of interest (ROI) were drawn in early and late time frames in the regions corresponding to the inferior vena cava (IVC) (Fig. 1A) and myocardium (Fig. 1B) respectively and time activity curves (TAC) generated. The blood TAC from IVC was first corrected for partial volume averaging by simulating the effect of convoluting a ~3-4 mm object (IVC) with a Gaussian distribution of FWHM spatial resolution of ~1 mm to generate recovery coefficients (RC). The blood TAC boosted by RC was then used in a 3-compartment kinetic model correcting for spill-out from the blood to the myocardium at the early time points and radioactivity recovery for the myocardium to compute rate of myocardial FDG influx, Ki(1/min). ROI was also drawn in the region corresponding to the brain (Fig. 1C) and cerebral FDG uptake rate, Ki(1/min), determined using computed input curve (Fig. 1D). Cardiac MRI was performed using the Bruker Clinscan 7T MR scanner on the same rats to assess LV structure and systolic function.

**Results:** A 1.8-fold increase in myocardial FDG Ki (Fig. 1E) was observed in non-failing SHR hearts (n=2) (Fig. 1F) when compared to control Wistar-Kyoto (WKY) hearts. There was, however, a significant reduction in myocardial FDG Ki (Fig. 1E) in failing SHR hearts (n=3) (Fig. 1F) with a significant increase in cardiac mass (Fig. 1G) when compared to WKY. Cerebral FDG Ki (Fig. 1E) was lower (~3.6-fold) in SHR than in WKY rats.

**Conclusions:** The pressure overload non-failing SHR heart enhances glucose metabolism to maintain cardiac function. The failing SHR heart, however, exhibits reduced glucose metabolism together with impaired function and significant LVH. Decreased heart function could result in decreased cerebral glucose metabolism and cerebral dysfunction.

**Acknowledgements**

NIH R01HL123627-03 (to BKK) and 1S10OD021672 (to SSB).

**References**

1. Brooks WW, Shen SS, Conrad CH, Goldstein RH, Bing OH: Transition from compensated hypertrophy to systolic heart failure in the spontaneously hypertensive rat: Structure, function, and transcript analysis. Genomics. 2010;95:84-92.

2. Zhong M, Kundu BK: Optimization of a Model Corrected Blood Input Function From Dynamic FDG-PET Images of Small Animal Heart In Vivo. IEEE Trans Nucl Sci. 2013;60:3417-3422.

3. González AJ, Aguilar A, Conde P et al: A PET Design Based on SiPM and Monolithic LYSO Crystals: Performance Evaluation. IEEE Trans Nucl Sci. 2016;63:2471-2477.

**Fig. 1 (abstract A7).**
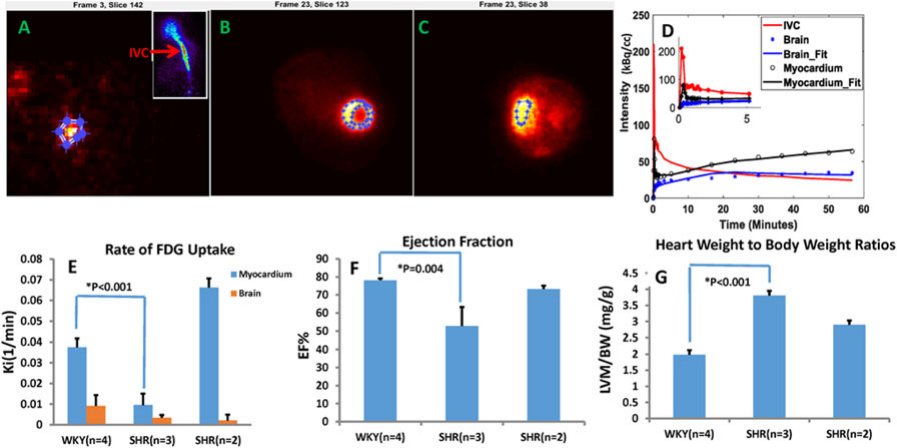
Total Body Dynamic FDG PET imaging. Regions of interest (ROI) shown on representative **(A)** Inferior vena cava (IVC) at early time point (inset: sagittal view), **(B)** Myocardium and **(C)** Brain transverse images at late time point from dynamic FDG PET images *in vivo*, **(D)** Time activity curves (TAC) shown for the blood (IVC) corrected by recovery coefficient, myocardium and the brain and model fits in a 3-compartment kinetic model (inset: TAC at early time points), **(E)** Computed rates of myocardial and cerebral FDG uptake, **(F)** Ejection fraction and **(G)** Heart weight to Body weight ratios measured using MRI *in vivo*. Student t-tests were performed only between WKY (n=4) and SHR (n=3)

### A8 TOFPET 2 based whole body PET

#### Ricardo Bugalho^1^, Luís Ferramacho^1^, Carlos Leong^1^, Tahereh Nikjnejad^2^, José C. Silva^1,2^, Rui Silva^1,2^, Miguel Silveira^1^, Stefaan Tavernier^2,3^, João Varela^1,2^

##### ^1^PETsys Electronics, Oeiras, Portugal; ^2^LIP, Lisbon, Portugal; ^3^Vrije Universiteit Brussel, Brussels, Belgium

###### **Correspondence:** Ricardo Bugalho

Small crystals with 1:1 coupling to SiPM yield the best time resolution for Time of Flight PET. However, applied to a whole body PET this results in a large number of electronics channels. A PET with 80 cm diameter and 2 meter axial length built on crystal and SiPM with a 3.2 mm pitch requires ≈512’000 electronics channels.

A system based on PETsys Electronics’ readout system is proposed. The elementary detector module consists an array of 16x16 LYSO crystals with a pitch of 3.2 mm, individually coupled to SiPM pixels and read by 4 64-channel TOFPET 2 ASICs. The system consists of 40 rings of 48 modules per ring, for a total of 1920 detector modules and a detector module power consumption of 5898 W. The modules are connected through cables to 120 FEB/D-4096 boards with Kintex-7 FPGAs, which provide power and bias voltage (from a single 12 V supply), configuration and readout to 16 detector modules each.

A central clock & trigger module provides synchronization for all the FEB/D (Fig. 1). It also supports a preliminary coincidence filter which discards non-coincident raw data in the FEB/D. The trigger geometry is fully configurable, allowing or forbidding coincidences between each pair of FEB/D. The FEB/D are connected to 4 DAQ PCIe cards through 16 daisy-chains of 6.6 Gbit/s optical links (7-8 FEB/D per chain; 4 links per PCIe card). The links carry both slow control and (raw) event data. Each link can transmit up to 100 Mcount/s and each DAQ PCIe card can transmit 250 Mcount/s to the computer for a total of 1 Gcount/s.

**Fig. 1 (abstract A8).**
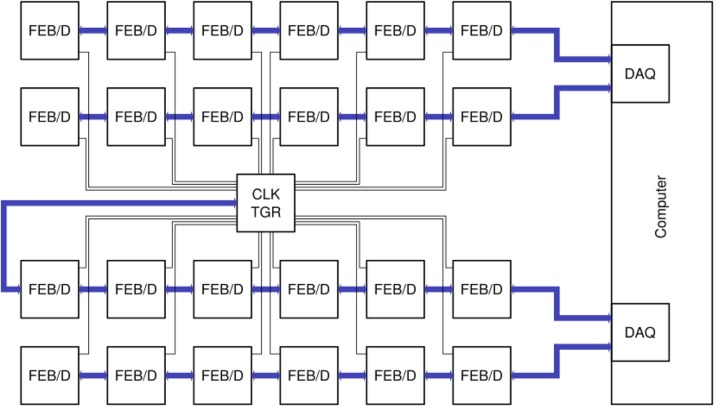
System topology

### A9 Mini-EXPLORER II: a prototype high-sensitivity PET/CT scanner for companion animal whole body and human brain scanning

#### Yang Lv^1^, Xinyu Lv^1^, Weiping Liu^1^, Martin S. Judenhofer^2,3^, Allison Zwingenberger^4^, Erik Wisner^4^, Sarah McKenney^2^, Edwin Leung^3^, Simon R. Cherry^2,3^ and Ramsey D. Badawi^2,3^

##### ^1^Molecular Imaging Business Unit, Shanghai United Imaging Healthcare, Co., Ltd., Shanghai, China; ^2^Department of Radiology, School of Medicine, University of California, Davis, CA, USA; ^3^Department of Biomedical Engineering, School of Engineering, University of California, Davis, CA, USA; ^4^Department of Surgery and Radiology, School of Veterinary Medicine, University of California, Davis, CA, USA

###### **Correspondence:** Yang Lv (yang.lv@united-imaging.com)

**Background**

The EXPLORER Consortium is developing a total-body PET scanner with high spatial resolution in collaboration with United Imaging Healthcare (Shanghai, China). As part of this program, we have designed and built MiniEXPLORER II which is expected to have high performance for companion animal whole body and human brain imaging. This scanner has been installed at the UC Davis School of Veterinary Medicine. Strategically, MiniEXPLORER II provides a system-level test-bench for the actual PET detectors, data acquisition system, data processing chain and acquisition software that will be used in the total-body EXPLORER scanner.

**Materials and methods**

The PET component has a ring diameter of 52 cm and an axial field of view of 48.3 cm. The detector modules are composed of arrays of LYSO crystals of dimensions 2.76x2.76x18.1 mm. Read-out is performed using 6x6 mm^2^ SensL J-series SiPMs. Anatomical information is provided by a CT scanner (uCT 510, United Imaging Healthcare). We have measured detector timing and energy resolutions. PET system performance was assessed using the NEMA NU2-2012 and NEMA NU4-2008 standards. Phantom studies were performed with the mini-Derenzo, the Hoffman brain and a long cylinder phantom. The first canine patient has been scanned (Fig. 1).

**Results**

System time-of-flight resolution was measured to be ~409 ps and average system energy resolution was ~11.7% at 511 keV. The NEMA NU2-2012 system sensitivity was found to be 55 kcps/MBq. Spatial resolution was 2.6 mm at 10 mm from the center of the FOV. 2.0 mm rods were clearly resolved on a mini-Derenzo phantom. Peak NEMA NU2-2012 NEC was 298.7 kcps at 8.4kBq/cc. System design and performance parameters are listed in Table 1.

**Conclusions**

We have constructed and characterized a fully-functioning high-sensitivity PET/CT scanner that has potential for human brain imaging, and whole body scanning in companion animals. The scanner has facilitated component and system-level prototyping for the first EXPLORER total-body PET scanner. Phantom and *in vivo* images have been acquired. Our next goal is to perform human brain studies on this system. We aim to begin such studies in the near future.

**Acknowledgements**

This work was supported in part by NIH Grant R01CA206187. We thank the Center for Imaging Sciences, School of Veterinary Medicine, UC Davis for technical support.

**Table 1 (abstract A9).** Design and performance parameters for mini EXPLORER II

Ring diameter	52cm
Axial FOV	48.3cm
Scintillator	LYSO
Crystal element size	2.76x2.76x18.1 mm^3^
Photodetector	SensL J-series SiPM
Timing resolution	409±39ps
Energy resolution	11.7%±1.5%
Energy window LLD	430 keV
Coincidence timing window	2.7ns (local section) and 2.9ns (inter-section)
NECR peak (NEMA NU4-2008)	1,762 kcps at 59kBq/cc
Scatter Fraction (NEMA NU4-2008)	17.8%
NECR peak (NEMA NU2-2012)	298.7 kcps at 8.4kBq/cc
Scatter Fraction (NEMA NU2-2012)	41.9%
Sensitivity (NEMA NU2-2012)	55.3 kcps/MBq at 0cm 59.0 kcps/MBq at 10cm

**Fig. 1 (abstract A9).**
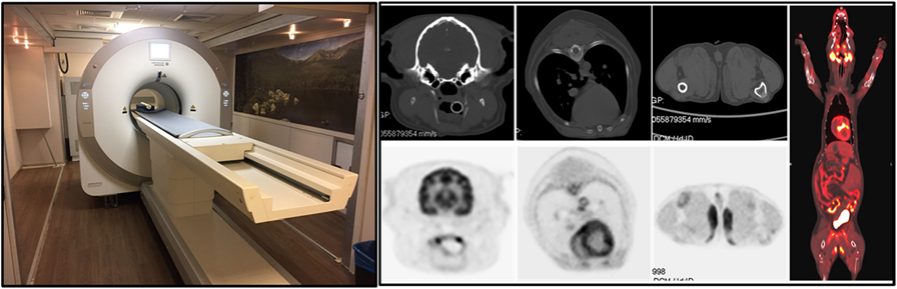
Mini-EXPLORER II. Left: As installed in UC Davis. Right: Images of the first canine patient

### A10 Optical simulation study for high resolution monolithic detector design for TB-PET

#### Mariele Stockhoff^1^, Roel van Holen^1,2^, Stefaan Vandenberghe^1^

##### ^1^MEDISIP, Ghent University, Ghent, Belgium, ^2^Molecubes, Ghent, Belgium

###### **Correspondence:** Mariele Stockhoff (mariele.stockhoff@ugent.be)

**Background**

The main limitations in positron emission tomography (PET) are the limited sensitivity and relatively poor spatial resolution. The administered radioactive dose and scan time could be reduced by increasing system sensitivity with a total-body (TB) PET design. The second limitation, spatial resolution, mainly originates from the specific design of the detectors that are implemented. In state-of-the-art scanners, the detectors consist of pixelated crystal arrays, where each individual crystal is isolated from its neighbors with reflector material. To obtain higher spatial resolution the crystals can be made narrower which inevitably leads to more inter-crystal scatter and larger dead space between the crystals.

A monolithic detector design shows superior characteristics in (i) light collection efficiency (no gaps), (ii) timing, as it significantly reduces the number of reflections and therefore the path length of each scintillation photon and (iii) spatial resolution (including better depth-of-interaction (DOI)). The aim of this work is to develop a precise simulation model based on measured crystal data and use this powerful tool to find the limits in spatial resolution for a monolithic detector for the use in TB-PET.

**Materials and methods**

A detector (Fig. 1) based on a monolithic 50x50x16 mm^3^ lutetium-(yttrium) oxyorthosilicate (L(Y)SO) scintillation crystal coupled to an 8x8 array of 6x6mm^2^ silicon photomultipliers (SiPMs) is simulated with GATE. A recently implemented reflection model for scintillation light allows simulations based on measured surface data (1). The modeled surfaces include black painted rough finishing on the crystal sides (16x50mm^2^) and a specular reflector attached to a polished crystal top (50x50mm^2^).

Maximum Likelihood estimation (MLE) is used for positioning the events. Therefore, calibration data is obtained by generating 3.000 photo-electric events at given calibration positions (Fig. 1). Compton scatter is not (yet) included. In a next step, the calibration data is organized in three layers based on the exact depth coordinate in the crystal (i.e. DOI assumed to be known). For evaluating the resolution, the full width at half maximum (FWHM) is estimated at the irradiated positions of Fig. 2 as a mean of all profiles in vertical and horizontal direction. Next, uniformity is evaluated by simulating 200k events from a flood source, placed in the calibrated area.

**Results**

For the irradiation pattern in Fig. 2 the resolution in terms of FWHM when applying MLE is: 0.86±0.13mm (Fig. 3a). Nevertheless, there are major artifacts also at non-irradiated positions. By positioning the events based on three DOI-based layers it can be seen that the events closest to the photodetector introduce the largest artifacts (Fig. 3b-d). The FWHM improves for Layer 1 and 2, to 0.69±0.04mm and 0.59±0.02mm, respectively. Layer 3 introduces major artifacts to the flood map, as events are positioned at completely different locations as the initial irradiation. A FWHM estimation is thus not useful.

The uniformity (Fig. 4) degrades with proximity to the photodetector. The map in Fig. 4c shows that the positioning accuracy depends not only on DOI but also the position in the plane parallel to the photodetector array.

**Conclusions**

A simulation model for a monolithic PET detector with good characteristics for TB-PET systems was developed with GATE. A first estimate of the spatial resolution and uniformity was given, pointing out the importance of depth-dependent effects. Future studies will include several steps towards more realistic simulations e.g. surface measurements of our specific crystals for the optical surface model and inclusion of the Compton effect.

**Reference**

1. Stockhoff M, Jan S, Dubois A, Cherry SR, Roncali E. Advanced optical simulation of scintillation detectors in GATE V8 . 0 : First implementation of a reflectance model based on measured data. Phys Med Biol. 2017;62:L1–8.

**Fig. 1 (abstract A10).**
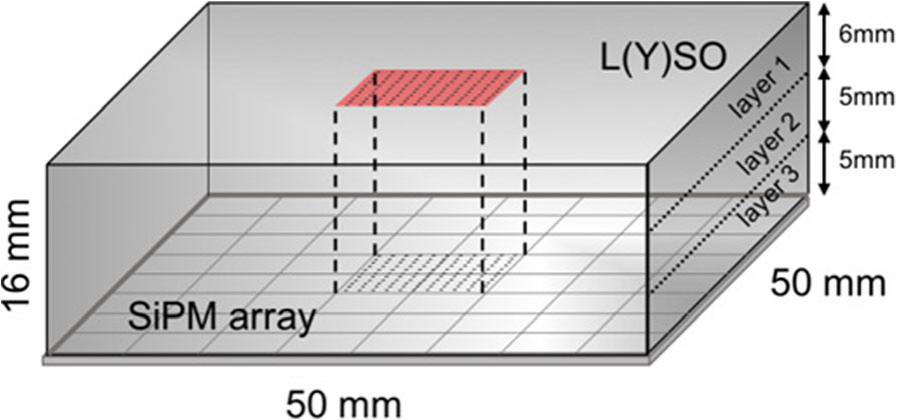
The detector is calibrated in a central area of 10x10mm at 11x11 positions

**Fig. 2 (abstract A10).**
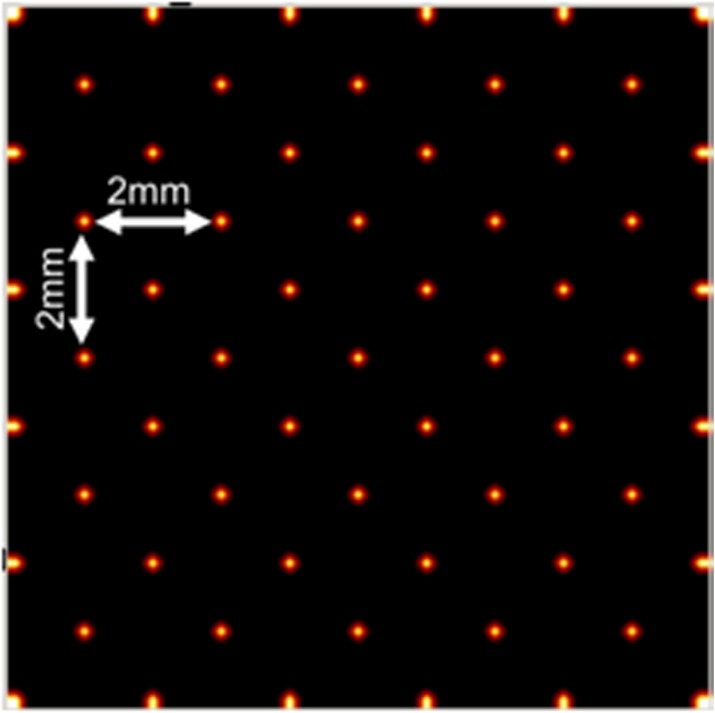
Irradiation pattern to obtain spatial resolution. Horizontal and vertical distance between points is 2mm

**Fig. 3 (abstract A10).**
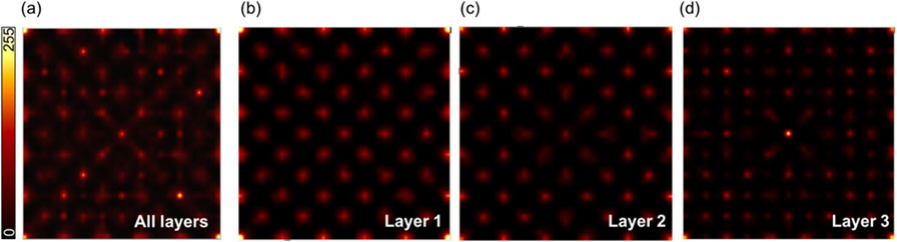
(a) Positioning without depth discrimination. In (b) - (d) events are positioned using three depth dependent calibration maps (with N=1k/layer)

**Fig. 4 (abstract A10).**
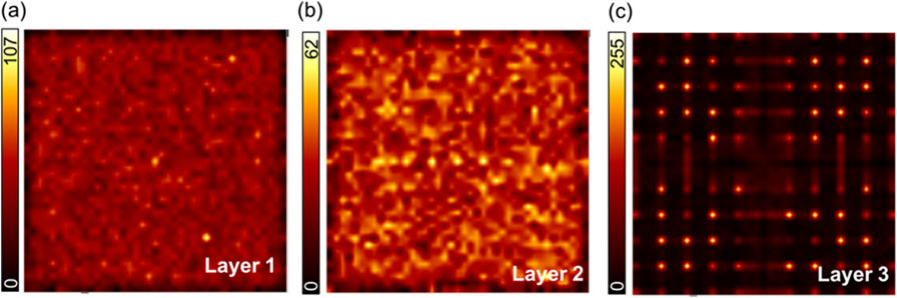
Flood maps of three depth dependent calibration maps (with N=1k/layer) with (a) 84651 counts (b) 61641 counts and (c) 53199 counts.The number of bins is 41x41

### A11 Time-efficient calibration enabled by a Gradient-Tree-Boosting-based positioning method for monolithic scintillators suitable for total-body PET

#### Florian Müller, David Schug, Patrick Hallen, Volkmar Schulz

##### ^1^Department of Physics of Molecular Imaging Systems, Institute for Experimental Molecular Imaging, RWTH Aachen University, Aachen, Germany

###### **Correspondence:** Florian Müller (florian.mueller@pmi.rwth-aachen.de)

**Background**

Monolithic scintillators are considered an alternative to segmented arrays [1-4]. Monoliths combine good energy and spatial resolutions at high sensitivity, offer intrinsic DOI-capabilities and are easier to fabricate, reducing the costs. This is especially important for total-body PET as a large number of detectors is required. To implement monoliths in total-body PET, an easy and time-efficient calibration is needed. Furthermore, an algorithm implementable in the system architecture for a large number of detectors is favorable. We present a time-efficient calibration and Gradient-Tree-Boosting (GTB)-based positioning algorithm reducing the calibration time to minutes per detector.

**Materials and Methods**

We employed PDPC’s technology evaluation kit as a coincidence setup and arrays of DPC 3200-22 sensors (tile) [5]. A white-wrapped 12 mm-high monolithic LYSO-scintillator matching the active tile-area of 32 mm was studied. Using a fan beam collimator with 0.25 mm slit and 199 Hz coincidence rate, we irradiated parallel lines at known positions independently along both planar directions.

We employed GTB to create regression models based on sequential binary decisions [6]. GTB models are implementable in FPGAs while data usage is the limiting aspect [7,8]. Previously, we proposed two scenarios to find the best possible positioning performance: One restricting data usage to enable an FPGA implementation and one without any restrictions.

GTB models were fit with training data with pitches ranging from 0.25-0.75 mm and varying the number of events per irradiation position. This enables reducing the required irradiation time which is linear to the number of irradiation positions and events. Unknown data of 0.25 mm pitch were employed for validation. We calculated positioning-error-distribution-based performance parameters for each irradiation position: a) SR: The distribution width (FWHM). b) r_90_: The radius enclosing 90 % of all events. c) Bias med.: The mean-positioning-error median.

**Results**

GTB models show a similar positioning performance for all studied calibration-grid pitches. Using 0.75 mm pitch reduces the calibration time by a factor 3 sacrificing less than 1.7 % of the positioning performance. Figure 1 shows performance parameters against irradiation time. Increasing the amount of training data improves the positioning performance. Only small improvements are observed after a sufficient amount of data is reached. All GTB models have a stable positioning performance without significant bias effects. An SR of less than 2 mm FWHM is reached for less than 5 min calibration time for both directions.

C**onclusion**

Calibration times of less than 5 min for monoliths and well-performing positioning models were achieved by significantly reducing the amount of required training data. This enables monoliths-based total-body PET with a large number of detector stacks.

**References**

[1] G. Borghi, V. Tabacchini, and D. R. Schaart, “Towards monolithic scintillator based TOF-PET systems: practical methods for detector calibration and operation,” *Phys. Med. Biol.*, vol. 61, no. 13, pp. 4904–4928, Jul. 2016.

[2] P. Bruyndonckx, S. Leonard, C. Lemaitre, S. Tavernier, and Y. Wu, “Performance Study of a PET Detector Module Based on a Continuous Scintillator,” *IEEE Trans. Nucl. Sci.*, vol. 53, no. 5, pp. 2536–2542, 2006.

[3] R. Marcinkowski, P. Mollet, R. Van Holen, and S. Vandenberghe, “Sub-millimetre DOI detector based on monolithic LYSO and digital SiPM for a dedicated small-animal PET system,” *Phys. Med. Biol.*, vol. 61, no. 5, p. 2196, 2016.

[4] A. González-Montoro *et al.*, “Detector block performance based on a monolithic LYSO crystal using a novel signal multiplexing method,” *Nucl. Instruments Methods Phys. Res. Sect. A Accel. Spectrometers, Detect. Assoc. Equip.*, Feb. 2018.

[5] C. Degenhardt *et al.*, “The digital silicon photomultiplier - A novel sensor for the detection of scintillation light,” *IEEE Nucl. Sci. Symp. Conf. Rec.*, pp. 2383–2386, 2009.

[6] F. Müller, “Evaluation and Calibration of Monolithic Scintillator Crystals for Positron Emission Tomography,” RWTH Aachen University, 2017.

[7] R. Kułaga and M. Gorg, “FPGA IMPLEMENTATION OF DECISION TREES AND TREE ENSEMBLES FOR CHARACTER RECOGNITION IN VIVADO HLS,” *Image Process. Commun.*, vol. 19, no. 3, pp. 71–82.

[8] B. Van Essen, C. Macaraeg, M. Gokhale, and R. Prenger, “Accelerating a Random Forest Classifier: Multi-Core, GP-GPU, or FPGA?,” in *2012 IEEE 20th International Symposium on Field-Programmable Custom Computing Machines*, 2012, pp. 232–239.

**Fig. 1 (abstract A11).**
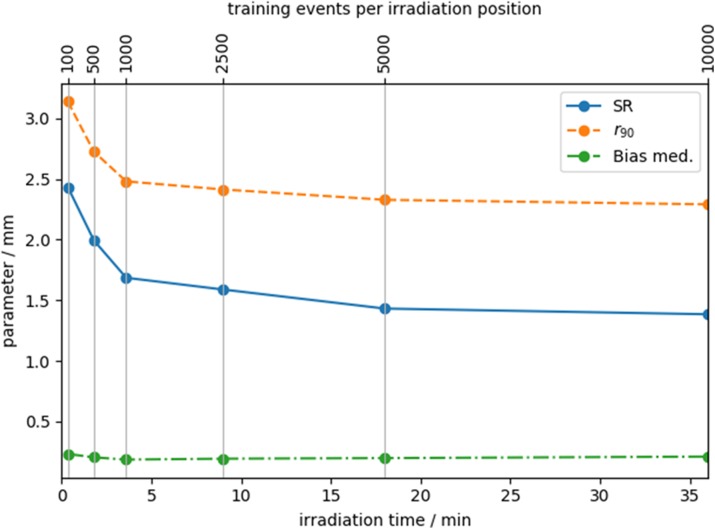
Performance parameter against training events per irradiation position (upper x-axis) and irradiation time (lower x-axis) for one calibration direction. Training data of 0.75 mm pitch were used. SR and r_90_ were averaged over the whole crystal. The data usage of the GTB models was not restricted during the training. All models required less than 2 MB memory

### A12 Initial performance assessment of a software-based coincidence processor for the EXPLORER total-body PET scanner

#### Edwin K. Leung^1,2^, Martin S. Judenhofer^1,2^, Simon R. Cherry^1,2^, Ramsey D. Badawi^1,2^

##### ^1^Department of Biomedical Engineering, University of California at Davis, Davis, California, 95616, USA; ^2^Department of Radiology, University of California at Davis Medical Center, Sacramento, California, 95817, USA

###### **Correspondence:** Edwin K. Leung (ekleung@ucdavis.edu)

**Background**

Coincidence processing in most positron emission tomography (PET) scanners is performed during data acquisition in real-time. However, on the EXPLORER total-body PET scanner we plan to store unprocessed single event (“singles”) data for later processing. This allows re-processing of the same singles dataset while varying parameters that could not otherwise be altered (e.g. energy and time windows).

Simulations have shown that EXPLORER can produce 6-7 times more singles data than shorter, clinical scanners at peak noise-equivalent count rates. Here we report an initial performance assessment of a software-based coincidence processor specifically developed for the EXPLORER to process the singles data at near real-time.

**Materials and methods**

Our coincidence processor was written in C++11 using standard C++ libraries and compiled with GCC 5.4 (as provided in the Ubuntu 16.04 LTS operating system). The framework of the coincidence processor is similar to the coincidence sorter featured in GATE v8.0.

The data for the performance assessment were simulated using GATE v8.0. Tables 1 and 2 provide the parameters used for the simulation parameters and for our coincidence processor. The data were then converted into a 64-bit list-mode data format for compactness.

The performance of the coincidence processor was evaluated using a Dell PowerEdge R730 rack server with dual Intel Xeon E5-2650 v4 CPUs. All data were stored in an Intel 750 series Peripheral Component Interconnect Express (PCIe)-attached solid state drive (SSD) (model SSDPEDMW012T4X1). The program execution time was measured using the C++ class std::chrono::steady_clock provided in C++11. We reset the file cache after every run to ensure that the data is read directly from the SSD and not from the cache.

**Results**

Figure 1 shows the coincidence processing performance of our coincidence processor using varying number of CPU threads (up to our expected 500 Mcps burst incoming singles data rates). Figure 2 shows the number of computer nodes needed to achieve near real-time coincidence processing.

**Conclusions**

We have developed a software-based coincidence processor that should have adequate performance to process list-mode singles data at near real-time provided that we have enough computer nodes. At a singles data rate of approximately 150 Mcps (equivalent to a 370-MBq injection evenly distributed in an adult phantom), this can be achieved with 2 computer nodes running dual Intel Xeon E5-2650 v4 CPUs.

**Acknowledgements**

This work was supported by NIH R01 CA206187, NIH R01 CA170874 and the UC Davis Research Investment in Science and Engineering program.

**Table 1 (abstract A12).** Simulation parameters

PARAMETER	
Crystal size	2.76 x 2.76 x 18.1 mm^3^
Crystal pitch	2.85 mm transaxial x 2.85 mm axial
# crystals per block	7 transaxial x 6 axial
# block detectors	120-block ring x 112 rings
Timing resolution	409 ps
Energy range	250 - 712 keV
Energy resolution	11.7% @ 511 keV
Dead time	none
Phantom	water-based cylindrical phantom (16 cm dia. * 150 cm long) with centered line source
Radioactive source	generic 511-keV back-to-back line source (6 mm dia. * 150 cm long)
Source activity	40 - 730 MBq
LYSO density	7.1 g/cm^3^
LYSO % by mass	Lu = 71.447%, Y = 4.034%, Si = 6.371% and O = 18.148%
LYSO background	400 Bq/cc
# detected singles simulated	approximately 2B singles for each singles data rate

**Table 2 (abstract A12).** Parameters used for the coincidence processor

PARAMETER	
Energy window	430 - 712 keV
Time window	fixed (8.0 ns) followed by variable (3.5 to 8.0 ns)
Transaxial FOV restrictions	686 mm
Axial FOV restrictions	restricted via the variable time window
Coincidence policy	Multiple Window, takeAllGoods

**Fig. 1 (abstract A12).**
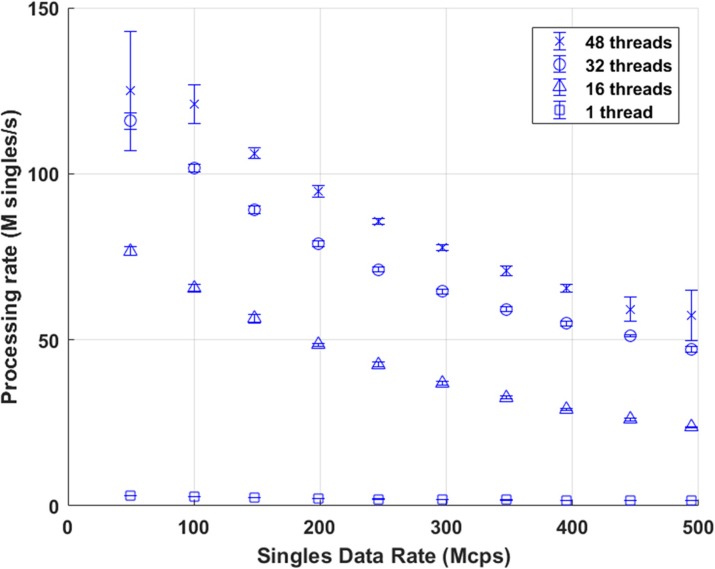
Performance of the coincidence processor. The error bar represents the standard deviation of 10 individual runs

**Fig. 2 (abstract A12).**
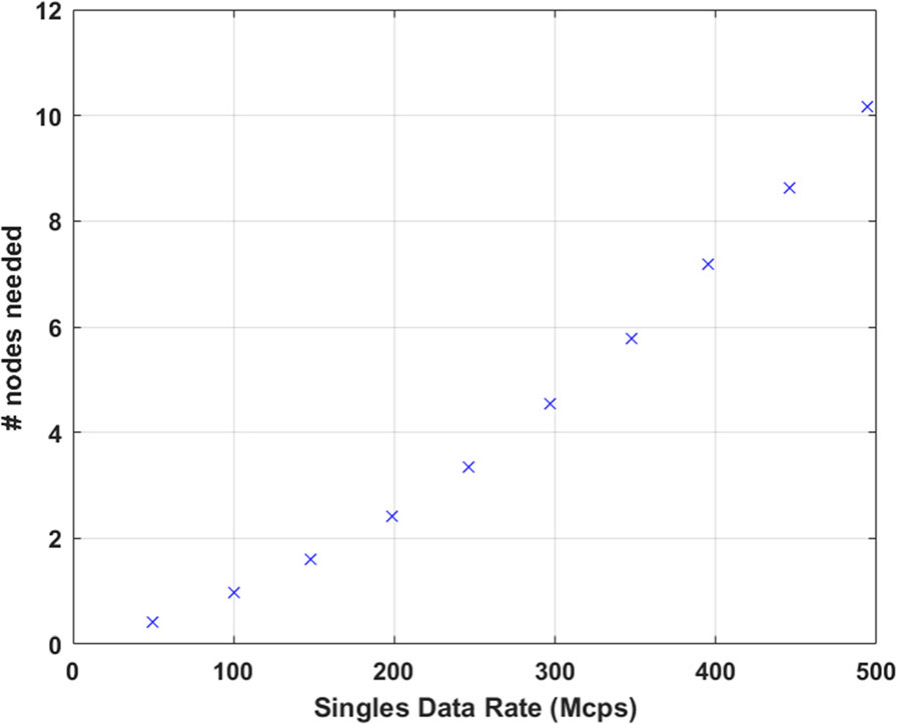
Number of computer nodes needed to achieve near real-time coincidence processing performance

### A13 Complementing Total Body PET by low dose Computed x-ray Tomography (CT) using photon counting, energy dispersive x-ray detectors

#### York Haemisch^1^, Christer Ullberg^2^, Alex Stewart^2^, Korbinian Mechlem^3^, Sebastian Ehn^3^, Thorsten Sellerer^3^, Peter Noel^4^, Julia Herzen^3^, Franz Pfeiffer^3^

##### ^1^Direct Conversion GmbH, Munich, Germany; ^2^Direct Conversion AB, Danderyd, Sweden; ^3^Technical University Munich, Dept. of Physics, Munich School of Bioengineering, Munich, Germany; ^4^Technical University Munich, Klinikum Rechts der Isar, Dept. of Radiology, Munich, Germany

###### **Correspondence:** York Haemisch

One of the strong arguments for Total Body PET is its better utilization of dose administered to the patient, thus enabling low dose studies as can be required in e.g. pediatrics or pregnant women.

Since total body PET devices will usually be complemented by an x-ray CT, the question of better dose utilization/dose reduction arises for the X-ray transmission tomographic imaging modality too.

Due to their better noise characteristics, the use of photon counting, energy dispersive x-ray detectors in (low dose) CT was first proposed a while ago [1-3] and detector developments such as MEDIPIX [4] have further sparked recent interest. The first experimental systems have been installed by major clinical imaging manufacturers [5-7]. Despite some initial difficulties, there seems to be consensus that this technology will play a major role in the future [8]. Over the last decade we have been developing hybrid photon counting, energy dispersive x-ray detectors based on a pixelated CdTe sensor layer and an CMOS-ASIC processing layer that are coupled 1:1 with a pixel dimension of 100 μm^2^ [9,10]. For their use in clinical CT, the ability to count at clinical photon flux rates (up to 10^9^ photons/mm^2^ s), as well as the correction of undesired effects such as charge sharing between pixels and hole cloud build-up in the detector material are essential features to enable high quality (clinical) imaging. The ability to determine energy levels by thresholding allows the elimination of very low energy counts, thus reducing noise levels and enabling significant dose reductions compared to integrating detectors [11]. Currently, our detectors provide measures to sort events into two energy bins; a next generation of detectors will allow sorting in up to six energy bins.

As is the case in emission tomography, X-ray CT image formation is performed in a processing chain, comprising detector, detector modelling, physical and geometric corrections, reconstruction and image representation [12]. With the transition from integrating to photon counting detection an adaptation of this chain is mandatory in order to obtain best image quality and to utilize the dose reduction potential. In particular, iterative reconstruction techniques are playing a crucial role in utilizing the full potential of the photon-counting mode [13]. The latest status of the technology will be demonstrated, and image examples from first (tomographic) photon counting devices in conjunction with iterative reconstruction techniques will illustrate the potential for dose reduction.

**References**

1. Shikhaliev O M, Xu T, Molloi S. Photon counting computed tomography: Concept and initial results. J. Med. Phys. 2005; 32: 427-436

2. Niederloehner D, Nachtrab F, Michel T, Anton G. Using the Medipix2 Detector for Photon Counting Computed Tomography. IEEE Nuclear Science Symposium Conference Record 2005; 2327-2331

3. Spartiotis K, Leppänen A, Pantsar T, Pyyhtiä J; Laukka P, Muukkonen K, Maennistoe O, Kinnari J, Schulman T. A photon counting CdTe gamma- and X-ray camera. Nucl. Instr. Meth. A 2005, 550, (1–2): 267-277

4. http://medipix.web.cern.ch/.

5. Kappler S, Henning A, Kreisler B, Schoeck F, Stierstorfer K, Flohr T. Photon counting CT at elevated X-ray tube currents: contrast stability, image noise and multi-energy performance. In: Whiting BR, Hoeschen C, eds. Proceedings of SPIE: medical imaging 2014 - physics of medical imaging. Vol 9033. Bellingham, Wash: International Society for Optics and Photonics, 2014; 90331C.

6. Schlomka JP, Roessl E, Dorscheid R, et al. Experimental feasibility of multi-energy photon-counting K-edge imaging in pre-clinical computed tomography. Phys Med Biol. 2008; 53(15): 4031–4047.

7. Zhang D, Li X, Liu B. Objective characterization of GE Discovery CT750 HD scanner: Gemstone spectral imaging mode. Med. Phys. 2011, 38 (3): 1178-1188

8. Taguchi K, Ivanczyk JS. Vision 20/20: Single photon counting detectors in medical imaging. Med. Phys. 2013, 40 (10): 100901-1 – 100901-19.

9. Ullberg C, Nilsson J, Weber N, Urech M, Lindman K, Lindqvist L, Engman A, Red A. Evaluation of the performance of a novel CdTe based photon counting detector for NDT applications. 2010 Conference: The forth Japan-US Symposium on Emerging NDE Capabilities published on: https://www.researchgate.net/publication/235423639_Evaluation_of_the_performance_of_a_novel_CdTe_based_photon_counting_detector_for_NDT_applications.

10. Ullberg C, Urech M, Weber N, Engman A, Redz A, Henckel F. Measurement of a dual-energy fast photon counting CdTe detector with integrated charge sharing correction. Proceedings of SPIE Medical Imaging 2013, Vol. 8668: 86680P-86680P-8.

11. Faby S, Kuchenbecker S, Simons D, Schlemmer HP, Lell M, Kachelrieß M. CT calibration and dose minimization in image-based material decomposition with energy-selective detectors. Proc. SPIE 9033, Medical Imaging 2014: Physics of Medical Imaging, 903318.

12. Dewey M, Kachelrieß M. Fundamentals of X-Ray Computed Tomography: Acquisition and Reconstruction. In: Sack I., Schaeffter T. (eds) Quantification of Biophysical Parameters in Medical Imaging. 2018, Springer, Cham

13. Mechlem K, Ehn S, Sellerer T, Braig E, Münzel D, Pfeiffer F, Noel P. Joint Statistical Iterative Material Image Reconstruction for Spectral Computed Tomography Using a Semi-Empirical Forward Model. IEEE Trans. Med. Imaging 2018, 37 (1): 68-80.

### A14 Design and performance study of a quasi-spherical PET scanner

#### David Perez-Benito^1^, Rigoberto Chil^1^, Jose Manuel Udías^2^, Manuel Desco^1,3,4^, Juan Jose Vaquero^1,3^

##### ^1^Universidad Carlos III de Madrid, Departamento de Bioingeniería e Ingeniería Aeroespacial, Leganes, Madrid, Spain; ^2^Universidad Complutense de Madrid, Grupo de Física Nuclear and UPARCOS, Madrid, Madrid, Spain; ^3^Instituto de Investigación Sanitaria Gregorio Marañón, Madrid, Madrid, Spain; ^4^Centro Nacional de Investigaciones Cardiovasculares (CNIC), Madrid, Madrid, Spain

###### **Correspondence:** David Perez-Benito

**Background**

Current challenges on new Positron Emission Tomography (PET) scanner design are high sensitivity, spatial and temporal resolution to accurately quantify dynamic biological processes. In this work, we present a proof of concept design and its figures of merit for a total-body mice PET. Simulated results about spatial sampling patterns, sensitivity and Noise Equivalent Count rate (NEC) are presented here.

**Methods**

The scanner shaped as an icosahedron built with 20 triangular facets of 155 mm side, covered by 10 hexagonal LYSO scintillator crystals (10 mm thick) coupled to a SiPM matrix (Fig. 1) [1]. The inscribed sphere has 234 mm diameter, and the field of view has been limited to 200 mm in diameter. Depth of Interaction information is obtained by engraving different layers in the crystal scintillator by means of Sub-Surface Laser Engraving [2]. The NEC curve has been simulated with a uniform distribution of ^18^F in a sphere of water (radius=20 mm) at the center of the FOV, dead time 200 ns, 100 ns pile-up, 2 ns coincidence time and 350-650 keV energy window. Simulations were done with GATE 7.2 and iterative image reconstruction was carried out directly for the root output. Sinograms were created for principal symmetries (those that define all the possible coincidences) to assess the spatial sampling sparsity. Resolution is being studied with a point source of ^18^F according to NEMA protocols.

**Results**

Representative sinograms are presented in Fig. 2 and correspond to all possible coincidence for a single symmetry in the upper cap and in the central section respectively, and they depict the range of visible angles with blind spots and fewer detected lines of response at the sides of the faces. The areas with higher sensitivity are the centers of the facets where there is direct coincidence with another facet in diametrical opposite direction. The high sensitivity due to the large solid angle coverage pushes the NEC knee far beyond typical scanners (3Mcps@85MBq). The estimated sensitivity is about 18% for an energy window of 350-650 keV. This arrangement results in a geometrical efficiency of 73% with respect to a 4π coverage.

**Conclusions**

This design shows favorable characteristics for fast, dynamic high-resolution total body PET imaging in mice, as well as the stringent requirements for the electronic readout of the SiPM arrays.

**Acknowledgement**

This work was partially funded by the Human Frontier Science Program (RGP0004/2013), projects RTC-2015-3772-1 and TEC2016-78052-R from the Spanish Ministerio de Ciencia e Innovación, and project TOPUS S2013/MIT-3024 from the regional government of Madrid. The research leading to these results has received funding from the Innovative Medicines Initiative (www.imi.europa.eu) Joint Undertaking under grant agreement n°115337, resources of which are composed of financial contribution from the European Union's Seventh Framework Program (FP7/2007-2013) and EFPIA companies in kind contribution. This work also acknowledges support by EU’s H2020 under MediNet, a Networking Activity of ENSAR-2 (grant agreement 654002).

**References**

[1] Perez-Benito D, R Herrera R, Chil R, Konstantinou G, Udías JM, Desco M, VaqueroJJ. Proposal for a PET scanner with 4π steradian span*.* Conference Record IEEE TNS-MIC Atlanta. 2017

[2] Konstantinou G, Chil R, Desco M, Vaquero, JJ. Sub-Surface Laser Engraving Techniques for Scintillator Crystals: Methods, Applications and Advantages. *IEEE Trans. Radiat. Plasma Med. Sci.* 2017; 1, 5:377-384.

**Fig. 1 (abstract A14).**
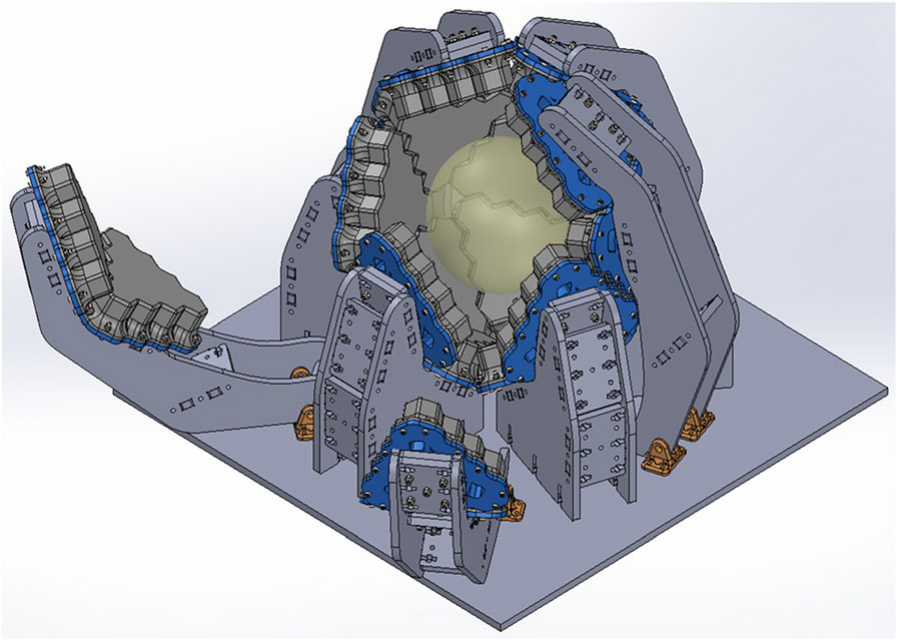
The scanner is formed by twenty modules arranged on ten sectors of two facets, half of them supported by hinges (orange); those sectors are moved by electromechanical actuators (not shown) to open the scanner. The yellow sphere represents the FOV

**Fig. 2 (abstract A14).**
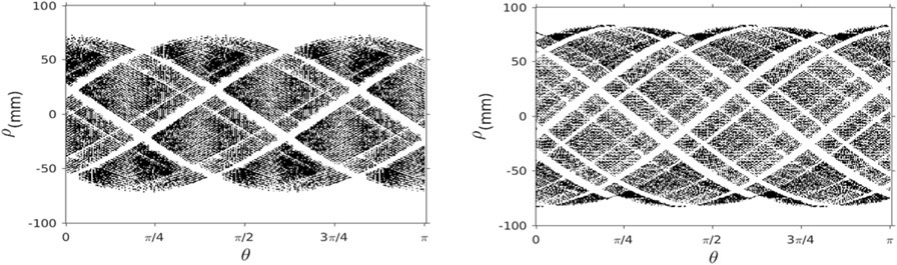
Sinograms of all possible combinations for two principal symmetries, the upper cap (left) and central section of the scanner (right). Slices correspond to heights z = 77mm and z = 22mm with respect to the center of the scanner

### A15 Flexible Geometry Iris_TOFPET Scanner

#### Stan Majewski (sm4aa@virginia.ed)

##### Department of Radiology and Medical Imaging Charlottesville, VA, 22903, USA

There is a big gap to bridge between the standard clinical whole body (WB) PET scanner with ~25 cm axial length coverage in one scanner position and the research EXPLORER type very large axial length (up to over 200 cm) scanners that are being developed. Both of these low- and high- end scenarios use the “one size fits all” approach and in the coming era of precision medicine it is not anymore an optimal or even acceptable approach. In most imaging protocols and imaging tasks there is potentially an unnecessary and even excessive use of radioactive imaging agents to compensate for the large diameter, highly suboptimal imaging geometry where a lot of the emitted radiation is not detected and resolution is not optimal, in order to accommodate all the cases that the one-universal-geometry-only system must include. In addition, while the rationale for the EXPLORER system is obvious to increase the sensitivity, this is a brute force approach, that will for example not provide optimal spatial resolution for many of the imaging tasks because the radius of these large scanners is fixed to allow for even the largest patients. Based on these considerations we are proposing a potential solution to enable filling the gap between these two “extreme” options while having in mind the performance and cost of these systems at the same time. Using the concept of sparsification we are able to propose a flexible adjustable arrangement of TOFPET detector modules that can be modified, depending on the requirements for the axial length coverage and scanner diameter to adapt to the individual imaging task, organ, and to the size of the patient. This reconfigurable system will be able to dynamically image from large volumes like torso/lungs using its 50 cm plus axial expansion to medium size organs like heart, kidneys, liver, prostate, etc using 25 cm option, and also can be adjusted to image head/neck, extremities, by using the most compact high spatial resolution and also 25 cm axial length configuration.

**Fig. 1 (abstract A15).**
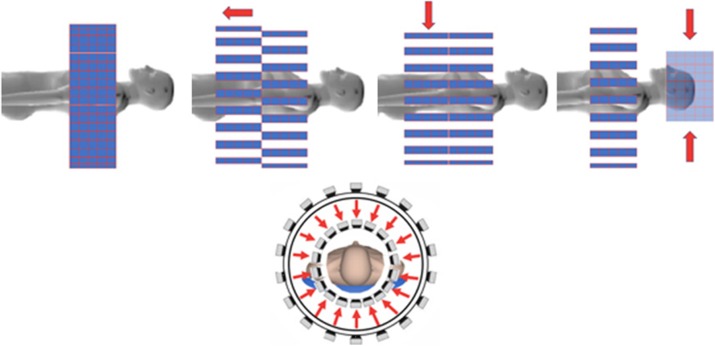
Illustration of the concept. The standard tight structure of 36 modules x4 rings with axial coverage of 25 cm gets separated into two sparse sections by an axial shift of about 25 cm. After angular rotation of 10 deg the two components form a uniform picket fence 50 cm long. As an example, after position adjustment in the axial coordinate one of the sectors gets compressed by factor ~2 to form high resolution and high sensitivity brain imager. Dynamic imaging of brain plus other organ(s) can be performed. Here is shown example of simultaneous dynamic heart-brain correlation imaging configuration. Other could be brain-spleen, brain-liver, etc

### A16 J-PET scanner combined with Positron Annihilation Lifetime Spectroscopy as a tool for morphometric imaging

#### Ewelina Kubicz^1^, Kamil Dulski^1^, Grzegorz Grudzień^2,3^

##### ^1^Faculty of Physics, Astronomy and Applied Computer Science, Jagiellonian University, S. Łojasiewicza 11, 30-348, Kraków, Poland; ^2^Department of Cardiovascular Surgery and Transplantology, Collegium Medicum, Jagiellonian University, Krakow, Poland; ^3^ John Paul II Specialist Hospital, Krakow, Poland

###### **Correspondence:** Ewelina Kubicz (ewelina.kubicz@doctoral.uj.edu.pl)

Jagiellonian Positron Emission Tomograph (J-PET) [1] is a multi-purpose detector which is used for investigations with positronium atoms in life-sciences as well as for development of medical diagnostics [2]. A prototype of the J-PET based on plastic scintillators was developed at the Jagiellonian University in Krakow, Poland [3,4].

Positron Annihilation Lifetime Spectroscopy (PALS) allows examining structure of materials at nano and sub-nanometer level. This technique is based on the lifetime and production intensity of ortho-positronium atoms in free volumes of given structures. It is mostly used for studies of organic materials.

J-PET tomograph is capable of imaging of properties of positronium produced inside the human body [2]. Thus far, there exist few results, e.g. by group of Y. C. Jean [5-6] and J-PET [7-8], showing that morphology of cells is correlated with the PALS parameters.

Results of the first experiments with the cardiac myxoma tumor conducted by the J-PET collaboration will be presented. We performed PALS studies with J-PET of well known structures such as XAD4 polymer and also first studies of human tissue (cardiac myxoma tumor and lipid mediastinal tissue). Results shown significance differences between tumor and normal tissue. As a result, we proved that PALS can be successfully used for studies of living organisms their dynamics and its relation to the cells morphology.

This result opens perspective for simultaneous determination of early and advanced stages of carcinogenesis by observing changes in biomechanical parameters between normal and tumour cells and standard PET examination.

**Acknowledgements**

This study is presented on behalf of the J-PET collaboration. We acknowledge the financial support by the National Science Centre through grants Nos. 2016/21/B/ST2/01222, 2017/25/N/NZ1/00861, the Ministry for Science and Higher Education through grants No. 6673/IA/SP/2016, 7150/E-338/SPUB/2017/1, the Foundation for Polish Science through TEAM programme.

**Ethics Approval**

The study was approved by Bioethical Commission of Jagiellonian University, approval number 1072.6120.123.2017

**Consent to publish**

Informed consent to publish has been obtained from this patient.

**References**

[1] Moskal P. et al. Time resolution of the plastic scintillator strips with matrix photomultiplier readout for J-PET tomograph**.** Phys. Med. Biol. 2016; 61:2025-2047.

[2] Moskal P. et al., Patent No: US 9851456; PL 227658; PCT/EP2014/068374.

[3] Moskal P. et al. A novel method for the line-of-response and time-of-flight reconstruction in TOF-PET detectors based on a library of synchronized model signals. Nucl. Instr. Meth. A. 2015; 775:54–62.

[4] Niedźwiecki S. et al. J-PET: A New Technology for the Whole-body PET Imaging. Acta Phys. Polon. B. 2017; 48(10):1567-1576

[5] Jean Y. C. et al. Applications of slow positrons to cancer research: Search for selectivity of positron annihilation to skin cancer. Applied Surface Science. 2006; 252:3166–3171.

[6] Liu G. et al. Applications of positron annihilation to dermatology and skin cancer. Phys. Stat. Sol. (c). 2007; 4(10):3912–3915.

[7] Jasińska B. et al. Human Tissues Investigation Using PALS Technique. Acta Phys. Polon. B. 2017; 48(10):1737-1747.

[8] Jasińska B. et al. Human Tissue Investigations Using PALS Technique - Free Radicals Influence. Acta Phys. Polon. A. 2017; 132:1556 – 1558.

### A17 Lesion Detection for Total-Body PET Imaging by Means of Deep Learning

#### Kuangyu Shi^1,2,3^, Lina Xu^2^, Giles Tetteh^2^, Andrei Gafita^3^, Matthias Eiber^3^, Andreas Buck^4^, Bjoern H Menze^2^, Axel Rominger^1^

##### ^1^Department Nuclear Medicine, University of Bern, Bern, Switzerland; ^2^Department Computer Science, Technical University of Munich, Munich, Germany; ^3^Department Nuclear Medicine, Technical University of Munich, Munich, Germany; ^4^Department Nuclear Medicine, University of Würzburg, Würzburg, Germany

###### **Correspondence:** Kuangyu Shi (kuangyu.shi@insel.ch)

**Background:**

Total-body PET provides an efficient imaging modality to extensively screen metastatic lesions and to assist the planning and monitoring of the emerging targeted radionuclide therapy. However, this powerful modality with valuable clinical information puts high demand in post-analysis. In this study, we aim to develop a computer-aided lesion detection method for total-body PET imaging. Here, it is extremely challenging considering the factor that dozens of lesions of heterogenous size and uptake may distribute in a variety of anatomical context with different background. We employ deep learning in this development, which extends human power of perception for information from data and has outperformed conventional machine learning methods in many applications.

**Materials & Methods:**

For proof-of-concept, we focus on the detection of bone lesions on whole body ^68^Ga-Pentixafor PET/CT imaging for patients with multiple myeloma. A fully convolutional neural network, called V-Net, was employed to detect and segment lesions. In addition, two V-Nets are cascaded to build a W-Net for the exploration of multimodal information of PET/CT for lesion detection. The algorithms were first tested on 70 digital phantoms generated by physically realistic simulation of ^68^Ga-Pentixafor PET images to demonstrate the applicability of deep learning methods. To assess the superiority of deep learning methods, we compared the proposed approach with several traditional machine-learning methods, including random forest, k-Nearest neighbor (kNN) classifier and support vector machine (SVM). Ultimately it was further evaluated on whole body PET/CT imaging of 12 patients with histologically proven primary multiple myeloma disease.

**Results:**

Compared with traditional machine learning methods, the V-Net has improved the precision from ~20% to 88.8% and the dice score from ~30% to 89.3% based on the test results on simulation phantoms. For the test on patient data, the V-Net achieved a sensitivity of 71.1%, and a specificity of 99.5%, PPV of 68% and NPV of 69.5%. The W-Net could slightly improve the performance to a sensitivity of 73.5%, and a specificity of 99.6%, PPV of 72.5% and NPV of 73.0%.

**Conclusions:**

We developed a computer-aided lesion detection for total-body PET based on deep learning. The test on a small number of datasets of ^68^Ga-Pentixafor PET/CT imaging has already shown improved accuracy compared to traditional machine learning methods. Increasing the amount of training data may further enhance the performance of the deep learning methods. In-depth test on a larger number of ^68^Ga-PSMA PET datasets is ongoing to further explore the clinical potential.

### A18 Comparison between total-body PET and conventional PET by means of Monte Carlo simulations in prostate cancer examinations

#### Charlotte Thyssen, Mariele Stockhoff, Stefaan Vandenberghe

##### Medisip, Ghent University, Ghent, Belgium

###### **Correspondence:** Charlotte Thyssen (thyssen.charlotte@gmail.com)

**Background**

Prostate cancer is by far the most common type of cancer in men. Prostate specific membrane antigen (PSMA) is a type II transmembrane protein which is highly overexpressed in prostate carcinoma. For this reason, PSMA is a good biomarker for the diagnosis of the disease. Therefore, a PSMA binding PET tracer is often used in prostate cancer examinations. The most widely used among these is the urea-based PSMA inhibitor Glu-urea-Lys-(Ahx)-HBED-CC (PSMA-11), linked to ^68^Ga. ^68^Ga is generated on site by means of a generator. Nowadays the activity generated daily is the limiting factor on the number of patients that can be scanned. The aim of this study is to validate by simulation if the increased sensitivity of TB-PET can be a solution to this clinical problem.

**Materials and methods**

A TB-PET system (with monolithic and pixelated block detectors, respectively) is compared to the current state-of-the-art PET scanner (GE discovery MI PET/CT). Figures 1 and 2 show the geometry of the scanners used for the simulations in the GEANT4 application for tomographic emission (GATE). First, the system sensitivity is evaluated according to the NEMA NU 2-2012 protocol. Secondly, the number of detected decays in the lesion is measured by simulating a 0.5 second scan for the conventional and the monolithic TB-PET design. An anthropomorphic model was created with XCAT and filled according to typical physiological tracer uptake [1] with 2MBq per kilogram bodyweight. The time between administration and scanning is one hour. The prostate in the model represents a high-grade prostate carcinoma.

**Results**

NEMA sensitivity of the GE discovery MI is 13.6 cps/kBq, matching information provided by GE. This sensitivity increases to 246.7 cps/kBq and 171.2 cps/kBq for a TB-PET with pixelated and monolithic crystals respectively. Per voxel inside the lesion, the TB-PET with monolithic crystal truly detected 1895.6±45.3 decays. The conventional system only detects 97.2±9.9 decays. The total number of true coincidences originating from the prostate lesion (indicated in Fig. 3) are 1,218,887 and 62,531 respectively. The TB-PET thus detected almost 20 times more decays originating from the lesion.

**Conclusions**

Since the number of detected decays inside the lesion is almost 20 times larger for TB-PET than for conventional PET, the amount of activity administered to the patient could be highly reduced. Due to this reduction, the number of patients that can be scanned daily can be increased accordingly. A TB-PET system would allow the clinicians to scan all patients using a single ^68^Ga generator.

**Reference**

[1] Afshar-Oromieh A, Malcher A, Eder M, Eisenhut M, Linhart H G, Hadaschik B A, Zechmann C M. PET imaging with a [^68^Ga]gallium-labelled PSMA ligand for the diagnosis of prostate cancer: biodistribution in humans and first evaluation of tumour lesions. Eur J Nucl Med Mol Imaging. 2013; 40(4): 486-495.

**Fig. 1 (abstract A18).**
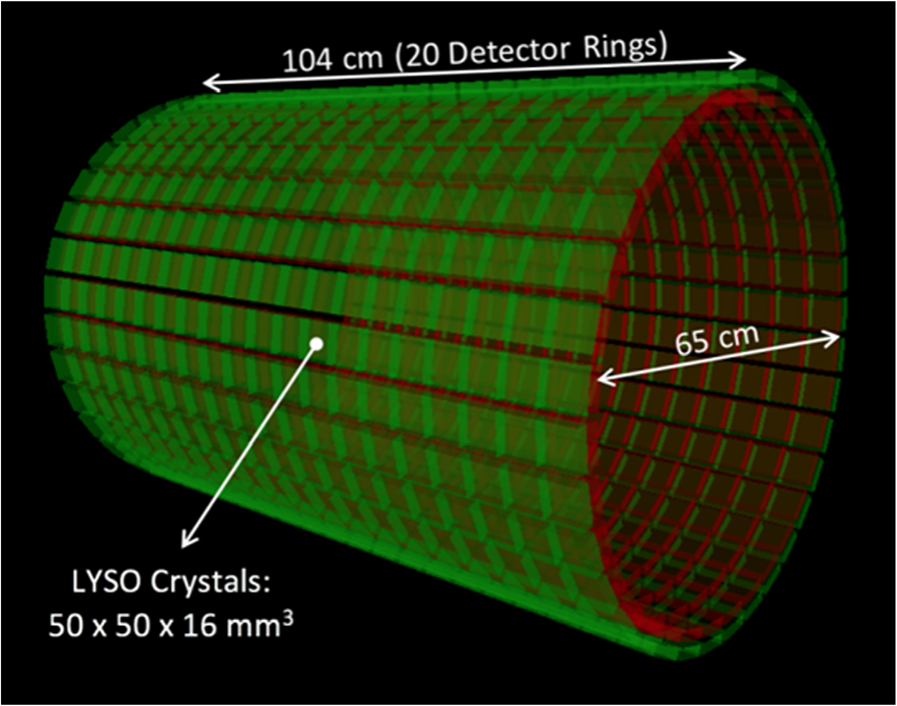
TB-PET with monolithic crystals

**Fig. 2 (abstract A18).**
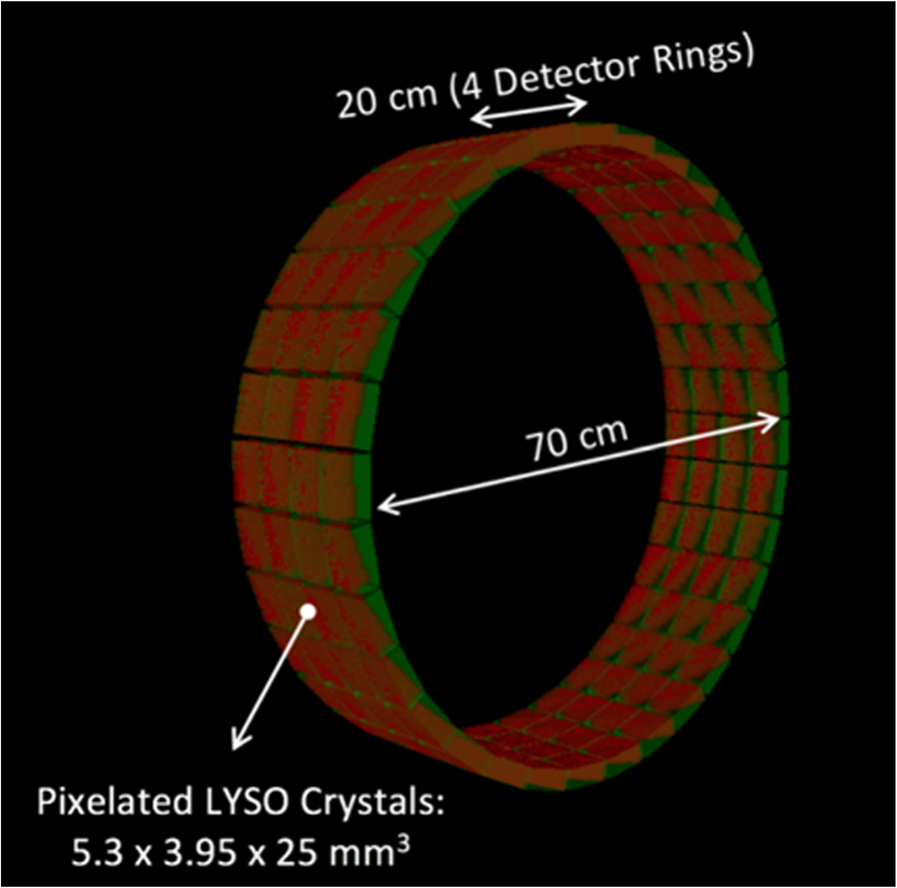
GE discovery MI PET/CT

**Fig. 3 (abstract A18).**
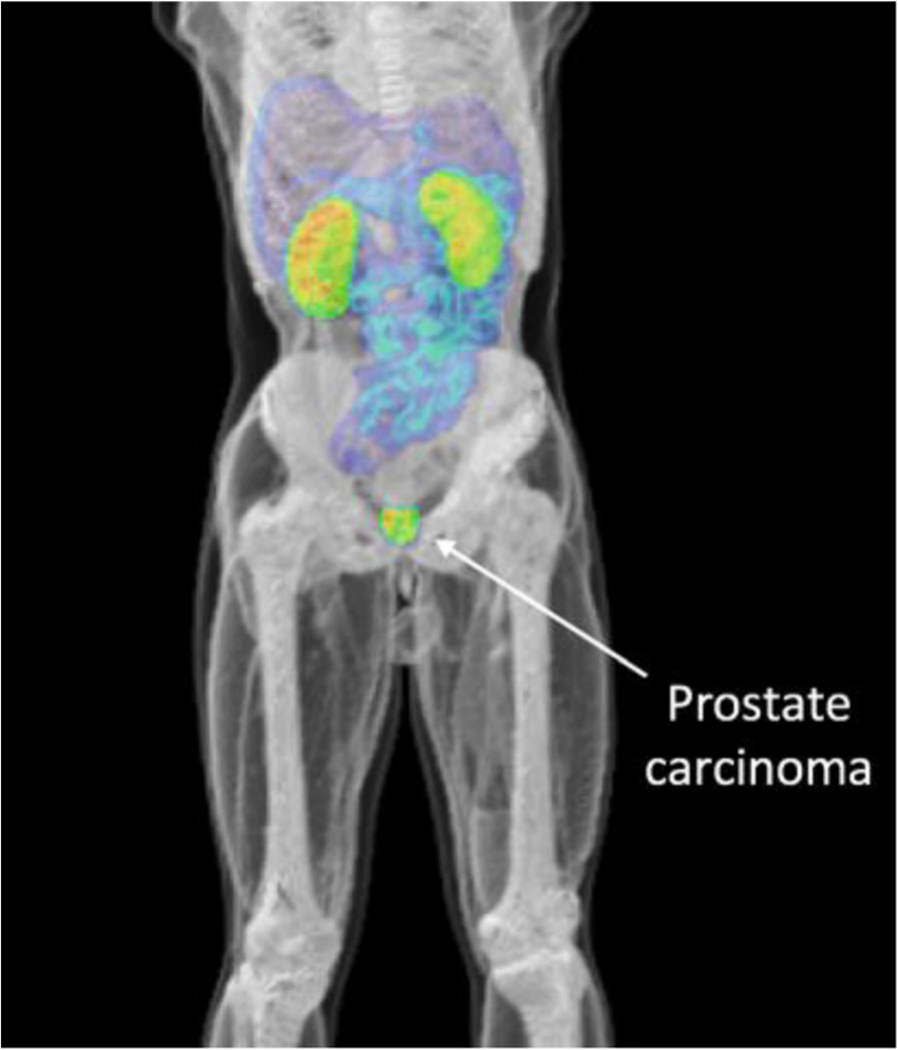
XCAT phantom with physiological tracer distribution and indication of prostate carcinoma

### A19 Development of a detector module with improved timing resolution for Time-of-Flight PET with single-sided readout and DOI capability

#### Tahereh Niknejad^1^, Ricardo Bugalh^2^, Agostino Di Francesco^1^, Luis Ferramacho^2^, Carlos Leong^2^, Manuel Rolo^3^, Rui Silva^1,2^, Jose Carlos Silva^1,2^, Miguel Silveira^2^, Stefaan Tavernier^2,4^, Joao Varela^1,2^

##### ^1^LIP, Lisbon, Portugal; ^2^PETsys Electronics, Oeiras, Portugal; ^3^INFN - sez. Torino, Italia; ^4^Vrije Universiteit Brussel, Brussels, Belgium

###### **Correspondence:** Tahereh Niknejad (tniknejad@lip.pt)

**Objectives**

In a total-body PET (TB-PET), depth of interaction (DOI) capability of the detector module is not very important because few gamma rays arrive at large angle relative to the axis of the crystals pixels. However, in TB-PET there will be annihilations where the two gamma rays are detected at large distance in the axial direction of the scanner especially in PET with long axial field-of-view (FOV), and these gamma rays arrive at a large angle relative to the axis of the crystals pixels. Most schemes to obtain DOI information ruin the time resolution, and are therefore not attractive for use in total body PET. In this work we present a simple approach allowing to obtain DOI without compromising the coincidence time resolution (CTR).

**Materials and methods**

The detector module consists an array of 4x4 LYSO crystals each 3.13x3.13x20 mm^3^ with a pitch of 3.2 mm, separated by Vikuiti foils, individually coupled to SiPM pixels and read by TOFPET2 ASIC [1]. The lateral surfaces of the crystal pixels are depolished and the light is shared among the pixels by having a glass plate on top of the crystal. This way, the amount of the light that is shared to the neighbour crystals gives an estimation of DOI [1,2].

However, the optical photons emitted from the crystal at different depths reach SiPM at different times depending on the path undergone inside the crystal. This photons’ propagation time degrades the CTR. Knowing DOI, it is possible to correct the timestamps provided by each channel of SiPM in order to improve the CTR as follows:

$$ {t}_i^{\prime }={t}_i-{\Delta }_i(DOI) $$

Where ${t}_i^{\prime }$ is the corrected time stamp, *t*_*i*_ is the initial time stamp and ∆_*i*_(*DOI*) is the delay in arrival time of the photons emitted at a certain DOI with respect to photons emitted at the back surface of the crystal.

**Results**

The distribution of DOI resolutions gives an average of 4.2 mm FWHM over 16 channels and an average of 3.8 mm FWHM over central channels. With an identical pair of modules in coincidence side-by-side (side irradiation) the average CTR over all channels after applying DOI correction improves from 464 ps FWHM to 299 ps FWHM. For top irradiation this improvement is from 356 ps FWHM to 291 ps FWHM.

**Conclusions**

These results demonstrate the feasibility of having a single-sided readout detector module with DOI capabilities and good timing performance for TOF application.

**References**

[1] Di Francesco A, Bugalho R, Oliveira R, et al. TOFPET2: a high-performance ASIC for time and amplitude measurements of SiPM signals in time-of-flight applications, J. Instrum. 2016 Mar;11(03):C03042.

[2] Pizzichemi M, Stringhini G, Niknejad T, et al. A new method for depth of interaction determination in PET detectors. Phys. Med. Biol. 2016 Jun;61(12):4679.

[3] Niknejad T, Pizzichemi M, Stringhini G, et al. Development of high-resolution detector module with depth of interaction identification for positron emission tomography. Nucl. Instr. Meth. Phys. Res. A. 2017 Feb;845:684-688.

### A20 Quantifying improved lesion detectability of the 70-cm PennPET Explorer using GATE simulations

#### Varsha Viswanath^1^, Suleman Surti^2^, Margaret E. Daube-Witherspoon^2^, Jeffrey P. Schmall^2^, Joel S. Karp^2^

##### ^1^Bioengineering Department, University of Pennsylvania, Philadelphia, PA, USA; ^2^Radiology Department, University of Pennsylvania, Philadelphia, PA, USA

**Background**

The increased sensitivity of a long axial field of view (AFOV) PET scanner allows for decreased patient dose or decreased scan time without loss of lesion detectability. Low dose and high throughput imaging will be important for adult as well as pediatric oncology studies, where patients under the age of 10 are routinely anesthetized when undergoing PET/CT (or PET/MR) scans to prevent motion. We are performing a lesion detectability study using Geant4 Application for Tomographic Emission (GATE) [1] simulations on the 70-cm PennPET Explorer [2] to determine the extent to which the scan time or dose can be reduced without compromising lesion detectability.

**Methods**

Using previously developed methodology [3], we initiated this study with a simulation of a 35-cm cylinder with 32 lesions (Fig. 1), to emulate an obese adult patient, on both the 70-cm PennPET Explorer scanner and a 23-cm scanner of similar geometry that represents a clinical PET system. Both 10-mm diameter spheres with a 4:1 activity ratio and 5-mm diameter spheres with a 6:1 activity ratio spheres were simulated to represent different conditions for lesion detection. The data were reconstructed using 3D list-mode time-of-flight (TOF) OSEM with all corrections applied [4]. The volume of interest (VOI) contrast results of the lesions were used to generate sphere and background probability distribution functions (PDFs) required to estimate lesion detectability using the method of scan statistics [5].

**Results**

The detectability of lesions is improved with the 70-cm PennPET Explorer scanner, compared to the 23-cm scanner, due to the increase in slice sensitivity. Images from a 30-s scan on the 70-cm scanner had similar visual detectability as those from a 3-min scan on the 23-cm scanner (Fig. 2). PDFs in Fig. 3, depicting histograms of contrast measurement for the 5-mm spheres on both scanners along with a background histogram, illustrate improved separation between background and sphere contrasts for the 70-cm scanner, compared to the 23-cm scanner. PDFs will be used to quantify the improvement in lesion detectability as a function of scanner geometry and scan time.

**Conclusions**

These methods will be translated to both pediatric and adult female XCAT phantoms (Fig. 4) [6] to quantify the improved lesion detectability of the 70-cm Explorer scanner when scan time or dose is reduced.

**Acknowledgments**

We acknowledge funding from NIH R01 CA206187, and NIH R01 CA113941.

**References**

1. Strulab D, Santin G, Lazaro D, Breton V, Morel C. GATE (Geant4 Application for Tomographic Emission): a PET/SPECT general-purpose simulation platform. Nuclear Physics B-Proceedings Supplements. 2003;125:75-9.

2. Viswanath V, Daube-Witherspoon ME, Schmall JP, Surti S, Werner ME, Muehllehner G, et al. Development of PET for total-body imaging Acta Physica Polonica B. 2017;48(10).

3. Surti S, Shore AR, Karp JS. Design study of a whole-body PET scanner with improved spatial and timing resolution. IEEE transactions on nuclear science. 2013;60(5):3220-6.

4. Popescu LM, Matej S, Lewitt RM, editors. Iterative image reconstruction using geometrically ordered subsets with list-mode data. Nuclear Science Symposium Conference Record, 2004 IEEE; 2004: IEEE.

5. Popescu LM, Lewitt RM. Small nodule detectability evaluation using a generalized scan-statistic model. Physics in Medicine & Biology. 2006;51(23):6225.

6. Segars W, Sturgeon G, Mendonca S, Grimes J, Tsui BM. 4D XCAT phantom for multimodality imaging research. Medical physics. 2010;37(9):4902-15.

**Fig. 1 (abstract A20).**
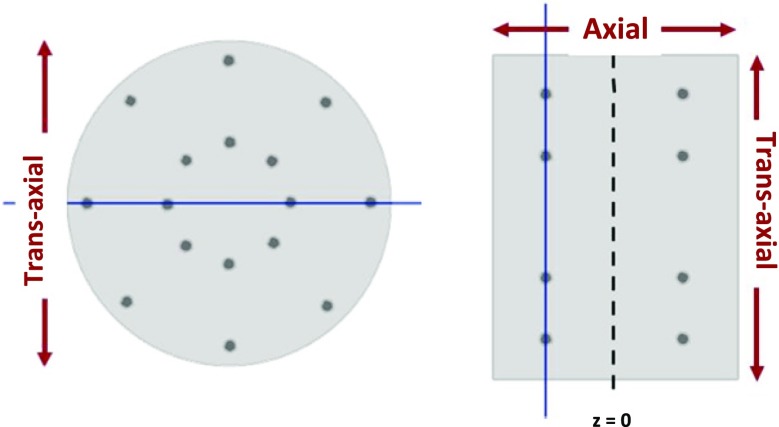
This figure shows the distribution of the 32 spheres in the cylinder. The cylinder was 35 x 23 cm (D x L) and had a background concentration of 0.1 *μ*Ci/cc. Sixteen spheres (eight each) were organized in an octagon at radii of 7 cm and 14 cm at z = ±5.75 cm with respect to the axial center (dashed black line)

**Fig. 2 (abstract A20).**
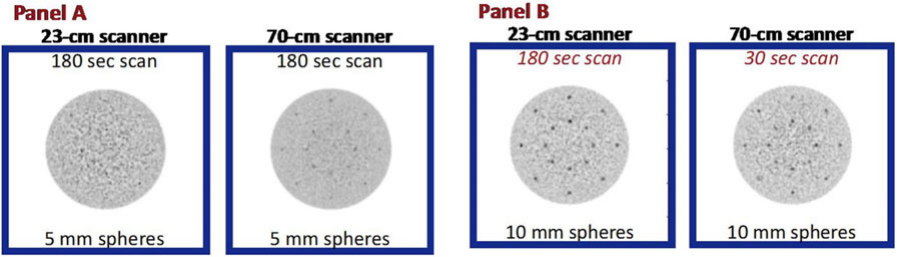
Panel A shows improved visual detectability of the 5-mm lesions on the 70-cm scanner (right) compared to the 23-cm scanner (left). Panel B shows similar visual detectability of the 10-mm lesions between a 3-min scan on the 23-cm scanner (left) and a 30-s scan on the 70-cm scanner (right). Because of the sensitivity gain with the longer AFOV, a 30-s scan on the 70-cm scanner has roughly the same number of events as a 180-s scan on the 23-cm system. All images are shown for the same iteration

**Fig. 3 (abstract A20).**
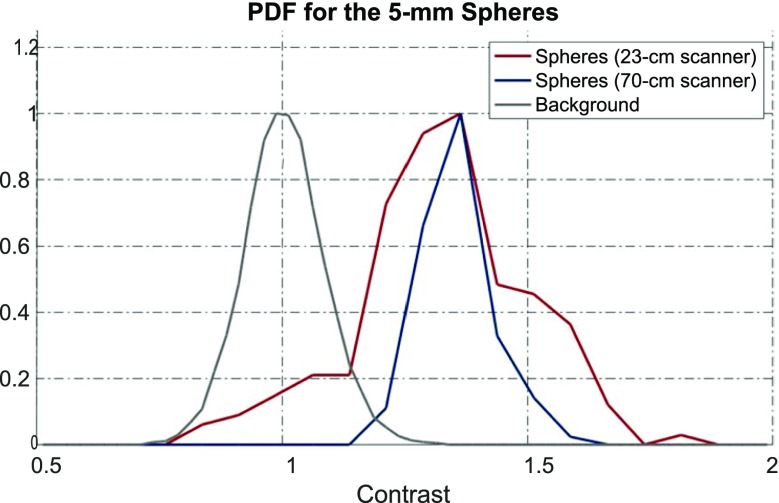
Probability distribution functions for the 5-mm lesions on the 23-cm scanner (red) and the 70-cm scanner (blue), along with the background PDF (grey), depicting less overlap between the signal and background PDFs for the 70-cm scanner compared to the 23-cm scanner and implying better lesion detectability. PDFs will be used to calculate lesion detectability using the scan statistics methodology

**Fig. 4 (abstract A20).**
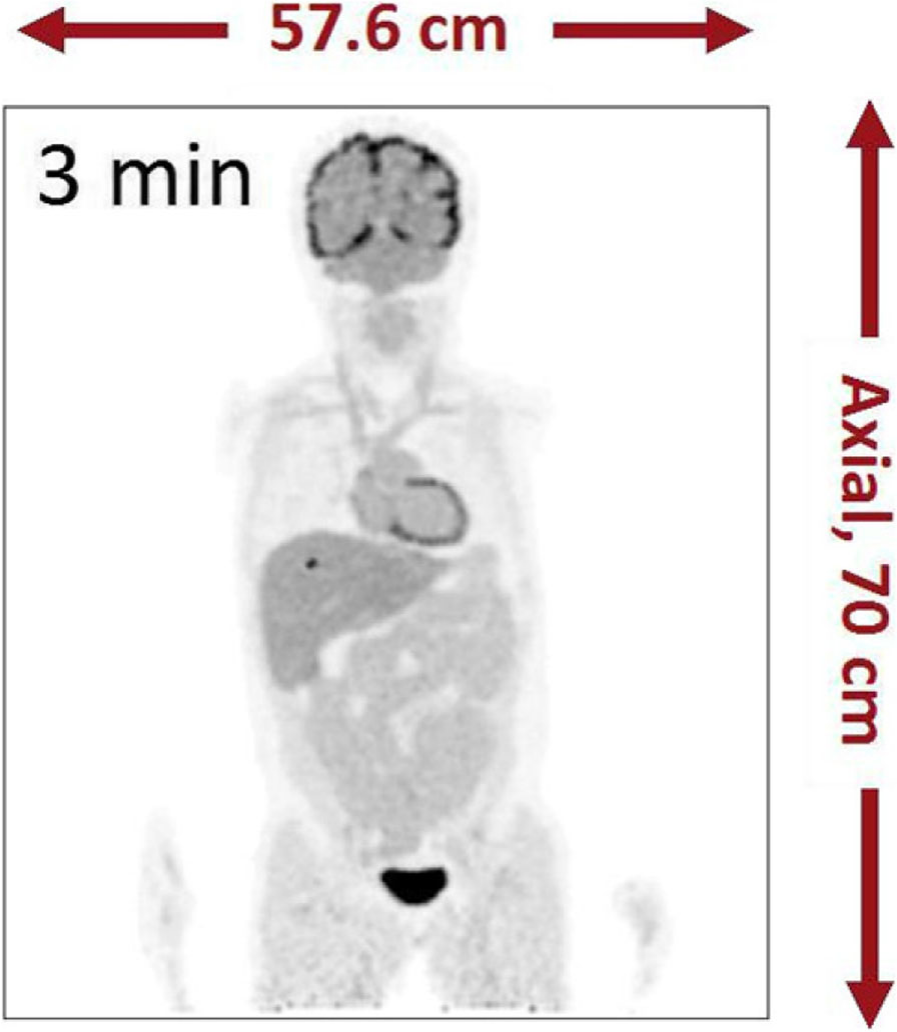
Image of the pediatric XCAT phantom simulated over a single bed position on the 70-cm PennPET Explorer scanner with a lesion in the liver. A scan covering this axial extent on the 23-cm scanner would require multiple bed positions. This phantom, along with an adult female phantom, will be used for future lesion detectability studies

### A21 Dynamic Imaging on the 70-cm PennPET Explorer using GATE Simulations

#### Varsha Viswanath^1^, Austin R. Pantel^2^, Margaret E. Daube-Witherspoon^2^, David A. Mankoff^2^, Joel S. Karp^2^

##### ^1^Bioengineering Department, University of Pennsylvania, Philadelphia, PA, USA; ^2^Radiology Department, University of Pennsylvania, Philadelphia, PA, USA

**Background**

A total-body PET scanner can dynamically image multiple lesions simultaneously. Additionally, the increased sensitivity of a long axial field-of-view (AFOV) scanner allows for improved accuracy of time activity curves, lower dose imaging, and finer temporal binning. To quantify the advantages of a total-body PET scanner for dynamic imaging, we simulated a dynamic fluorothymidine (FLT) study on the 70-cm PennPET Explorer [1], compared it to a 23-cm clinical scanner. We estimate the flux constant (K_FLT_), which has been shown to correlate with Ki-67 values, a cellular marker of proliferation [2, 3].

**Methods**

We used GATE [4] to model the 70-cm PennPET Explorer scanner and a 23-cm AFOV scanner with similar detector performance and geometry. Based on clinical FLT data [3], kinetic parameters were selected to emulate low, medium, and high K_FLT_ lesions (ml/cm^3^/sec) as well as a blood input curve. Data were used to fill the modified NEMA IEC phantom (10 and 13 mm spheres, Fig. 1). The list-mode data from a 60 min scan were then parsed into 45 frames (5s-300s frames), and then bootstrapped into twenty replicates to estimate the precision of kinetic parameters. Data were reconstructed using list-mode TOF OSEM [5], and all corrections were applied. Volumes of interest were drawn over all spheres and the lung insert and corrected for scan duration, decay, and partial volume effects. Data were fit in PMOD using a 2-compartment model, and the accuracy and precision of the K_FLT_ was calculated.

**Results**

Initial results show that the 70-cm scanner has greater precision of the time activity curves when compared to the 23-cm scanner, with greater impact in shorter time frames (Fig. 2). When comparing K_FLT_, accuracy and precision of the measurement improves by a factor of 2-3x with the 70-cm scanner, especially for the smaller spheres. For example, at high flux, the percent error for the 10-mm lesions improved from 23 ± 33% to 3.1± 12% for the larger AFOV scanner (Table 1).

**Conclusions**

This work provides preliminary data on the impact of improved sensitivity of the 70-cm PennPET Explorer on dynamic imaging. Future studies will quantify the effects of finer temporal sampling and lower dose injections using the XCAT phantom with embedded lesions at multiple locations. These studies will help to optimize imaging protocols that we plan to perform with the PennPET Explorer once operational.

**Acknowledgments**

We acknowledge funding from NIH R01 CA206187, NIH R01 CA113941, NIH R01 EB023274, and NIH R33-CA225310.

**References**

1. Viswanath V, Daube-Witherspoon ME, Schmall JP, Surti S, Werner ME, Muehllehner G, et al. Development of PET for total-body imaging Acta Physica Polonica B. 2017;48(10):1555-66.

2. Muzi M, Mankoff DA, Grierson JR, Wells JM, Vesselle H, Krohn KA. Kinetic modeling of 3′-deoxy-3′-fluorothymidine in somatic tumors: mathematical studies. Journal of Nuclear Medicine. 2005;46(2):371-80.

3. Muzi M, Vesselle H, Grierson JR, Mankoff DA, Schmidt RA, Peterson L, et al. Kinetic analysis of 3′-deoxy-3′-fluorothymidine PET studies: validation studies in patients with lung cancer. Journal of Nuclear Medicine. 2005;46(2):274-82.

4. Strulab D, Santin G, Lazaro D, Breton V, Morel C. GATE (Geant4 Application for Tomographic Emission): a PET/SPECT general-purpose simulation platform. Nuclear Physics B-Proceedings Supplements. 2003;125:75-9.

5. Popescu LM, Matej S, Lewitt RM, editors. Iterative image reconstruction using geometrically ordered subsets with list-mode data. Nuclear Science Symposium Conference Record, 2004 IEEE; 2004: IEEE.

**Fig. 1 (abstract A21).**
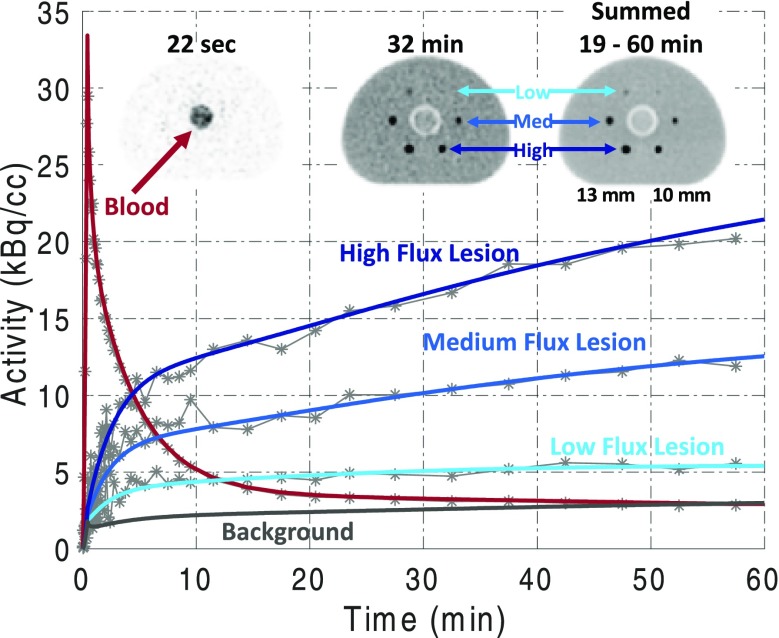
Ideal time activity curves for the blood, background, and lesions, with corresponding data curves shown in light grey (∗). The blood curve was derived from a small patient population and was used to fill the lung insert (μ = water) of the simulated NEMA IEC phantom (top-left). The low, medium, and high flux curves were generated using kinetic parameters from literature [3] and were used to fill the 10- and 13-mm lesions (top right). The uptake in the background was based on data on muscle uptake

**Fig. 2 (abstract A21).**
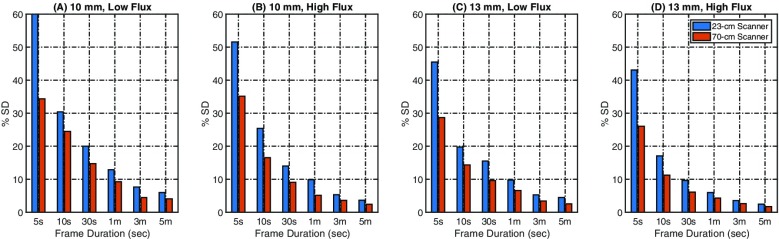
Percent standard deviation across 20 replicates of activity uptake in four lesions when simulated on the 23-cm and 70-cm scanners. 13-mm spheres (C, D) show improved precision compared to 10 mm spheres (A, B) and high flux lesions (B, D) show improved precision when compared to low flux (A, C) lesions. Precision data is grouped by frame duration, where there were 45 frames total (16x5s, 7x10s, 5x30s, 5x60s, 5x180s, 7x300s), to show the average precision per scan duration

**Table 1 (abstract A21).** Bias ± s. d. values of measured flux across 20 replicates using PMOD. Results show improved accuracy of flux (*K*1 ∗ *k*3/(*K*1 + *k*2)) and precision for the 70 cm scanner by a factor of 2-3 x

	Flux Bias ± s. d.					
	10 mm Sphere			13 mm Sphere		
	Low (%)	Med (%)	High (%)	Low (%)	Med (%)	High (%)
23 cm	-51 ± 30	12 ± 41	23 ± 33	-23 ± 53	4 ± 36	21 ± 20
70 cm	-19 ± 11	-3 ± 13	-3 ± 12	-12 ± 31	10 ± 5	4 ± 10

### A22 Monte Carlo study of a compact total body PET with monolithic scintillators

#### Maya Abi Akl^1,2^, Stefaan Vandenberghe^1^, Othmane Bouhali^2^, Yassine Toufique^3^

##### ^1^Department of Electronics and information systems, Ghent University, Ghent, Belgium; ^2^Science Program, Texas A&M University at Qatar, Doha, Qatar; ^3^Department of medical imaging, Mohammed VI University of health sciences, Casablanca, Morocco

###### **Correspondence:** Maya Abi Akl (Maya.AbiAkl@UGent.be)

**Background and aim**

Total body PET (TB-PET) is an emerging and promising concept in the field of medical imaging. It differentiates from the existing state-of-the-art whole-body PET scanners by a significant increase in body coverage by detectors. The proposed design has an axial field of view (FOV) of about 1 m considered to be the maximum standard sitting height of a human which will enable the most important organs of the body to be imaged simultaneously. In this work, the long axial TB-PET is simulated using the Geant4 application for tomographic emission (GATE) simulation package.

**Material and methods**

The scanner design consists of 36 detector modules per ring, and each module is made up of 50mm x 50mm x 16mm monolithic LYSO crystals. Different axial lengths of the scanner are considered starting at 5 cm, the length of one ring, up to 104 cm, the length of 20 rings repeated in the axial direction with a gap of 0.2cm. The absolute sensitivity is studied for a point source but also for more realistic distributions like cylindrical air and water phantoms of different lengths. The results are shown in Table 1. Also Scatter Fraction (SF) results for all three distributions are shown in Table 2.

**Conclusion**

Adding rings increases the sensitivity for all three distributions but clearly largest gains are seen for the extended sources with and without attenuation and so the increase in sensitivity is mostly effective for the long phantom. Also, very small changes in SF are observed for the long phantom with increasing number of rings.

**Table 1 (abstract A22).** Absolute sensitivity for point source, short phantom and long phantom in (a) air and (b) water

	(a) Absolute sensitivity		
Number of rings	Point Source in 100 cm air phantom	20 cm air phantom	100 cm air phantom
4	2.03	1.81	0.815
8	6.06	5.82	2.97
12	10.14	10,01	5.98
16	13.62	13.38	9.37
20	16.27	16.22	12.63
	(b) Absolute sensitivity		
Number of rings	Point Source in 100 cm water phantom	20 cm water phantom	100 cm water phantom
4	0.96	0.65	0.125
8	1.63	1.83	0.49
12	2.04	2.84	0.99
16	2.23	3.69	1.58
20	2.34	4.20	2.23

**Table 2 (abstract A22).** Scatter Fraction for point source, short phantom and long phantom in water

	Scatter Fraction (%)		
Number of rings	Point Source in 100 cm water phantom	20 cm water phantom	100 cm water phantom
4	24	26	28
8	29	27	30
12	32	27	30
16	33	26	30
20	34	26	30

### A23 Total-body dynamic imaging and late time-point antibody imaging in rhesus monkeys using the mini-EXPLORER scanner

#### Eric Berg^1^, Elizabeth Li^1^, Xuezhu Zhang^1^, Simon Williams^2^, Alice F. Tarantal^3^, Ramsey D. Badawi^1,4^, Simon R. Cherry^1,4^

##### ^1^Department of Biomedical Engineering, University of California-Davis, Davis, CA, USA; ^2^Department of Biomedical Imaging, Genentech Inc., South San Francisco, CA, USA; ^3^Department of Pediatrics and Cell Biology, California National Primate Research Center, University of California-Davis, Davis, CA, USA; ^4^Department of Radiology, University of California-Davis, Sacramento, CA, USA

**Background:** Two benefits of long axial field-of-view (FOV) PET imaging include total-body kinetic modeling to study multi-organ interactions, and using the increased sensitivity to extend the temporal dynamic range for measuring late time-point biodistribution and pharmacokinetics. Here we present two nonhuman primate imaging studies with the mini-EXPLORER system that demonstrate the feasibility of these applications.

**Methods:** The mini-EXPLORER scanner, a 45 cm axial FOV system based on Siemens mCT detectors [1], was used for all imaging studies. Images were reconstructed using a listmode time-of-flight OSEM algorithm that includes all corrections except scatter correction.

First, we performed a total-body dynamic study of ^11^C-raclopride to image dopamine D_2_ receptor binding simultaneously in the brain and gut. A healthy rhesus monkey was injected with ~94 MBq ^11^C-raclopride and imaged in a single bed position for 90 minutes. Time activity curves were manually extracted using AMIDE software.

Second, we performed a 30-day longitudinal imaging study of ^89^Zr-labeled antibodies to investigate the suitability of three chelators for long duration immuno-PET studies. Humanized monoclonal antibodies against the herpes simplex viral protein gD were labeled with ^89^Zr via one of three chelator-linker combinations. Nine healthy male rhesus monkeys were used to compare the pharmacokinetics of each chelator. Each monkey was injected with ~37 MBq ^89^Zr-antibody along with 10 mg/kg dose of unlabeled antibody and imaged six times over a 30-day period.

**Results:** A selection of ^11^C-raclopride images at different time points demonstrate excellent image quality and the ability to visualize uptake throughout the body with a single bed position (Fig. 1a). Time activity curves show high uptake in the liver and kidneys, and similar uptake for the striatum and gut (Fig. 1b).

Excellent image quality was also obtained in the ^89^Zr-antibody study (Fig. 2). Even at 30-days post-injection, representing ~9 half-lives of ^89^Zr and a total residual activity of only ~20 kBq, the image quality was sufficient to readily identify the signal in the liver, kidneys and joints. Significant differences in late time-point liver uptake and whole-body clearance were observed between the three chelators, while little variation (+/- 10%) was observed within each chelator group (Table 1).

**Conclusions:** The imaging studies presented here demonstrate the use of long axial FOV PET for total-body dynamic imaging and enabling late time-point imaging, up to 30-days post-injection with ^89^Zr-labeled antibodies. Future studies include kinetic modeling of the ^11^C-raclopride dataset along with comparing assay-based and image-based pharmacokinetics in the ^89^Zr-antibody study.

**Reference**

1. Berg E, Zhang X, Bec J, et al. Development and evaluation of mini-EXPLORER: a long axial FOV PET scanner for nonhuman primate imaging. J Nucl Med. 2018; in press.

**Fig. 1 (abstract A23).**
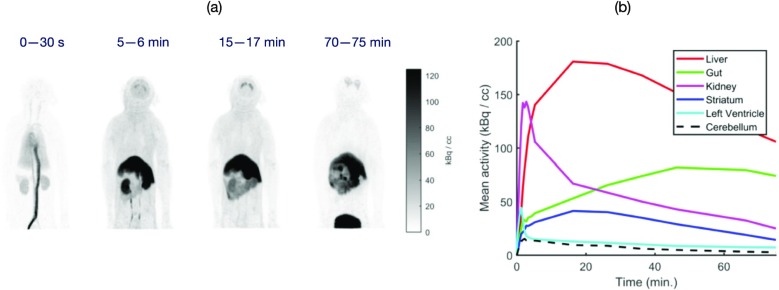
(**a**) Maximum intensity projection images at various time-points. (**b**) Time activity curves up to 75 minutes post-injection, representing approximately 4 half-lives of ^11^C

**Fig. 2 (abstract A23).**
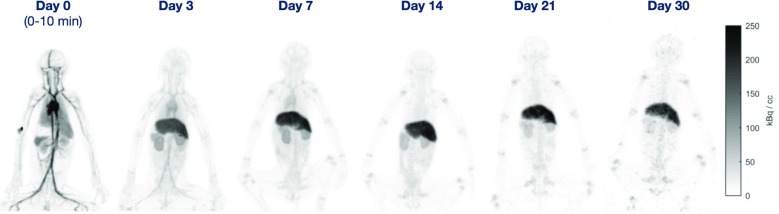
Maximum intensity projection images at each time-point for one of the nine monkeys. The monkey was injected with 43.6 MBq of ^89^Zr-labeled antibody approximately three minutes before the start of the day 0 scan

**Table 1 (abstract A23).** Quantification of decay-corrected activity in the liver and whole body for all animals at day 14 or day 17. All animals were administered ~37 MBq activity on day 0. There is little variation in liver uptake and overall clearance within each group, but significant differences are observed between the three groups

	*Group 1: day 17*			*Group 2: day 14*			*Group 3: day 14*		
Animal	A	B	C	D	E	F	G	H	I
Liver (MBq)	7.2	6.4	6.5	2.3	2.9	3.4	10.5	12.2	12.2
Whole body (MBq)	16.1	14.8	15.9	5.9	6.0	6.6	28.7	31.4	29.6

### A24 Feasibility of simultaneous dual-radionuclide time-of-flight PET imaging with PennPET Explorer

#### Stephen C. Moore^1^, Varsha Viswanath^2^, Scott D. Metzler^1^, Joaquín L. Herraiz^3^, Joel S. Karp^1^

##### ^1^Department of Radiology, University of Pennsylvania, School of Medicine, Philadelphia, Pennsylvania, 19104, USA; ^2^Department of Bioengineering, University of Pennsylvania, School of Engineering and Applied Sciences, Philadelphia, Pennsylvania, 19104, USA; ^3^Department of the Structure of Matter, Complutense University of Madrid, Madrid, Spain, 28040

###### **Correspondence:** Stephen C. Moore (scmoore1@pennmedicine.upenn.edu)

**Background**

We described previously a new approach for simultaneously imaging a conventional positron emitter and another radionuclide that emits a prompt gamma along with positron(s). This permits detection of triple-coincidence events that provide the key for separating the two tracers. In our approach, multiple-coincidence list-mode acquisition is necessary; however, no additional detectors are required beyond the PET ring, and the prompt gamma can be detected within the standard energy window around 511 keV. The goal of this study is to extend our method to time-of-flight (TOF) PET and to evaluate its expected performance by computer simulation of the PennPET Explorer system with a range of axial fields of view (AFOV) up to 70 cm.

**Methods**

Our fast Monte Carlo simulation used ray-tracing of annihilation photons and 602-keV prompt gammas from ^124^I (with β+ yield/decay = 0.227). Annihilation lifetime, attenuation, and detection (250-ps TOF resolution) were modeled stochastically; however, scatter was included only implicitly using a broad-beam attenuation coefficient (μ=0.07 cm^-1^) and a scatter fraction (0.286) obtained from GATE simulations. We evaluated count rates for each scanner length by simulating, separately for ^18^F and ^124^I, a 70-cm line source located 5-cm off-axis within a 20.4-cm-diameter cylindrical attenuator. Random double- and triple-coincidence rates were computed from simulated singles and doubles rates. A 70-cm-long NEMA IEC phantom was also simulated; the four smallest spheres contained ^18^F, while the two largest spheres contained ^124^I. Background water activity was included to provide sphere-background concentration ratios of 4:1 for each tracer. The total activity in the phantom during a 2.5-minute acquisition was 212 MBq for both ^18^F and ^124^I. Histo-images were reconstructed by backprojection, using double- and triple-coincidence efficiencies to separate ^124^I and ^18^F.

**Results**

The ^18^F and ^124^I true-plus-scatter coincidence rates were the same for equal positron-emission rates (Fig. 1), while the average randoms rate for ^124^I was 7.1 times greater than that of ^18^F, providing noise-equivalent count rates (NECR) ~37% higher for ^18^F than for ^124^I. The NECR for ^124^I and ^18^F both increased by factors of 3.9 and 8.3, respectively, for 46- and 70-cm AFOV, compared to 23-cm AFOV. IEC phantom images (Fig. 2) demonstrated excellent separation of ^18^F and ^124^I, and reduced image noise for the longer AFOVs.

**Conclusion**

Simultaneous dual-radionuclide TOF PET imaging is expected to perform well for the PennPET Explorer, which will have an axial FOV of 70 cm or greater (up to 140 cm) and 250-ps TOF resolution.

**Acknowledgement**

The authors are grateful for support from the U.S. National Institutes of Health, under grant R01-CA206187.

**Fig. 1 (abstract A24).**
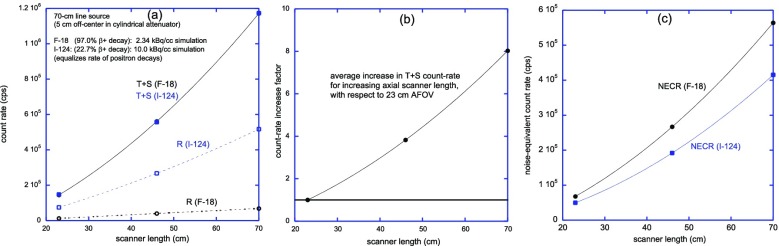
Simulated count-rate performance for a 70-cm-long NEMA line source, containing either ^18^F or ^124^I and positioned 5 cm off-center within a 20.4-cm cylindrical attenuator, for 23-, 46-, and 70-cm axial field-of-view (AFOV) lengths corresponding to 1, 2, and 3 detector rings of the PennPET Explorer. Activity of each tracer was adjusted to yield equal rates of positron decays. **(a)** True + scatter (T+S) count rates (solid curves) and random (R) count rates (dashed curves) for ^18^F (black) and ^124^I (blue). T+S count rates for ^124^I were the same as those from ^18^F for equal positron-decay rates. ^124^I random rates (~7.1 x higher than for ^18^F) included double- and triple random events. **(b)** The count rates for 2 and 3 detector rings vs. a single ring increased appropriately with detection solid angle for this off-center source, when photon angles of incidence on the detector were restricted to be within ±45-degrees of normal incidence. **(c)** The resulting NECR for ^124^I and ^18^F for each system. The NECR for both ^124^I and ^18^F increased by factors of 3.9 and 8.3, respectively, for 2 and 3 detector rings, compared to a single-ring system

**Fig. 2 (abstract A24).**
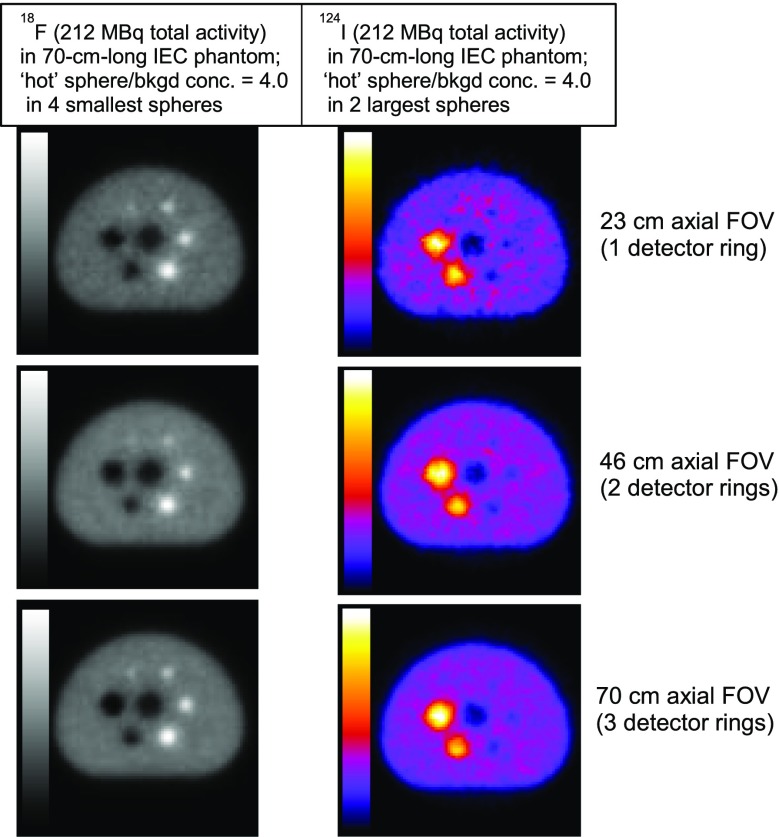
^18^F and ^124^I TOF histo-images, obtained by backprojection of attenuation-corrected histo-projections. The three image rows from top to bottom correspond to simulated 2.5-minute simultaneous acquisitions of both tracers using, respectively, 1, 2, and 3 axial PennPET Explorer detector rings, showing progressively reduced image noise for the longer PET systems. The ^124^I images in the right-hand column were made solely from triple-coincidence events. The ^124^I histo-projections were scaled, using the known efficiencies for double- and triple-coincidence events, and then subtracted from the total double-coincidence histo-projections to estimate the TOF data for ^18^F alone. The resulting ^18^F histo-images are shown in the left-hand column. These separated histo-images will be used to initialize a simultaneous joint reconstruction of both tracer distributions from all recorded double- and triple-coincidence events using OSEM

### A25 Design of brain PET with DOI method for Alzheimer’s disease

#### Minju Lee^1^, Kilyoung Ko^1^, Youngtaek Kim^2^, JungYeol Yeom^2^, Gyuseong Cho^1^

##### ^1^Department of Nuclear and Quantum Engineering, Korea Advanced Institute of Science and Technology, Deajeon, KS015, Republic of Korea; ^2^Department of Bio-Covergence Engineering, Korea University, Seoul, KS013, Republic of Korea

###### **Correspondence:** Minju Lee (mjlee8695@kaist.ac.kr); Gyuseong Cho

Positron Emission Tomography (PET) is widely used for cancer screening, heart disease, brain disease, and etc. Alzheimer’s disease, known as the most common form of dementia, has several competing hypotheses to explain the cause of the disease. The amyloid hypothesis postulated that the major reason of Alzheimer’s disease is due to precipitation of extracellular amyloid beta (Aβ) [1]. This disease can be diagnosed by measuring the distribution of beta amyloid plaques using PET. PET for the whole body has the advantage of being able to inspect large objects, but it is expensive and has a low sensitivity. Optimized brain PET for diagnosis of Alzheimer’s disease is needed. The purpose of this study is to design of brain PET with depth of interaction (DOI) for diagnosis of Alzheimer’s disease using Monte Carlo simulation. If the length of the scintillation crystal is long, a parallax error occurs in which two gamma rays generated at a distance from the origin are obliquely incident on the scintillation crystal. This error makes the spatial resolution non-uniform and can be corrected by measuring DOI. The spatial resolution of brain PET should be uniform because beta amyloid plaques are more distributed in the outside than in the center of the brain. Many DOI measurement techniques have been developed. DOI techniques, based on sharing and redirection of scintillation light among multiple detectors and together with attenuation of light over the length of the crystals were used for this study [2]. Figure 1 shows DOI techniques. The overall dimensions of LYSO were 24 x 24 x 15 mm3, each 3 x 3 x 15 mm3, and each lateral faces were depolished for DOI. The Geant4 Application for Tomographic Emission: a simulation toolkit for PET and SPECT was used this study to design of brain PET. Brain PET consisted of detector module, ring detector, signal processing board, data acquisition board, reconstruction algorithm, and software. Detector module and ring detector consisted of two sets of 8x8 array LYSO coupled silicon photomultiplier and 72 modules, respectively. The inner diameter and axial length of brain PET were 293.5 mm and 51 mm, respectively. Radioactive source emitting 511 keV gamma rays was located at the center and outside of detector ring. Uniformity of spatial resolution, energy resolution, and sensitivity will be evaluated.

**References**

1. J. hardy, D. Allsop. Amyloid deposition as the central event in the aetiology of Alzheimer’s disease. TiPS. 1991;Vol. 12:383-388

2. M. Pizzichemi et al. A new method for depth of interaction determination in PET detectors. Phys. Med. Biol. 2016;Vol 1:1-20

**Fig. 1 (abstract A25).**
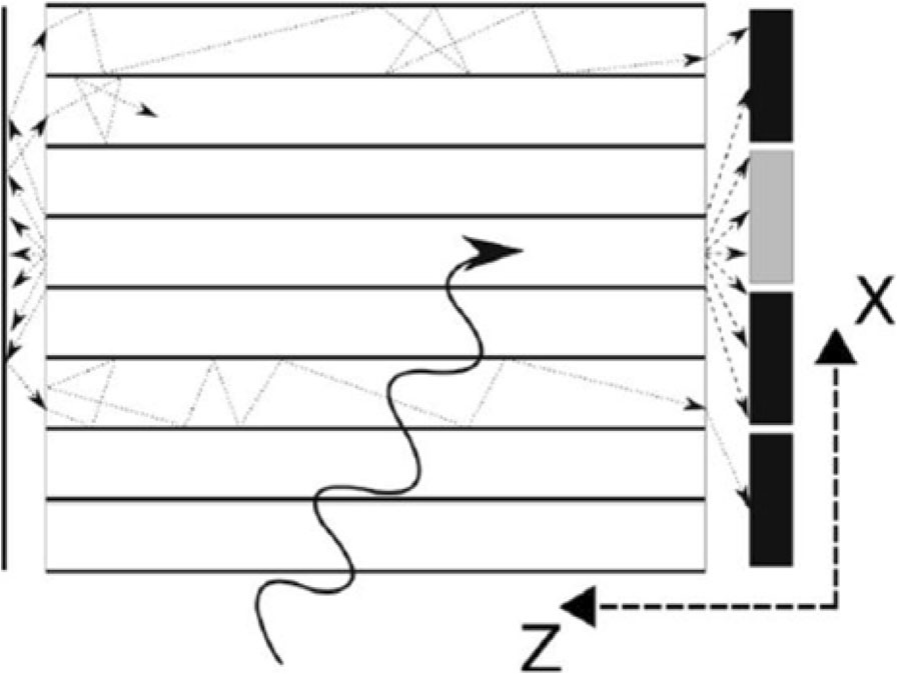
Schematic of the proposed DOI method

**Fig. 2 (abstract A25).**
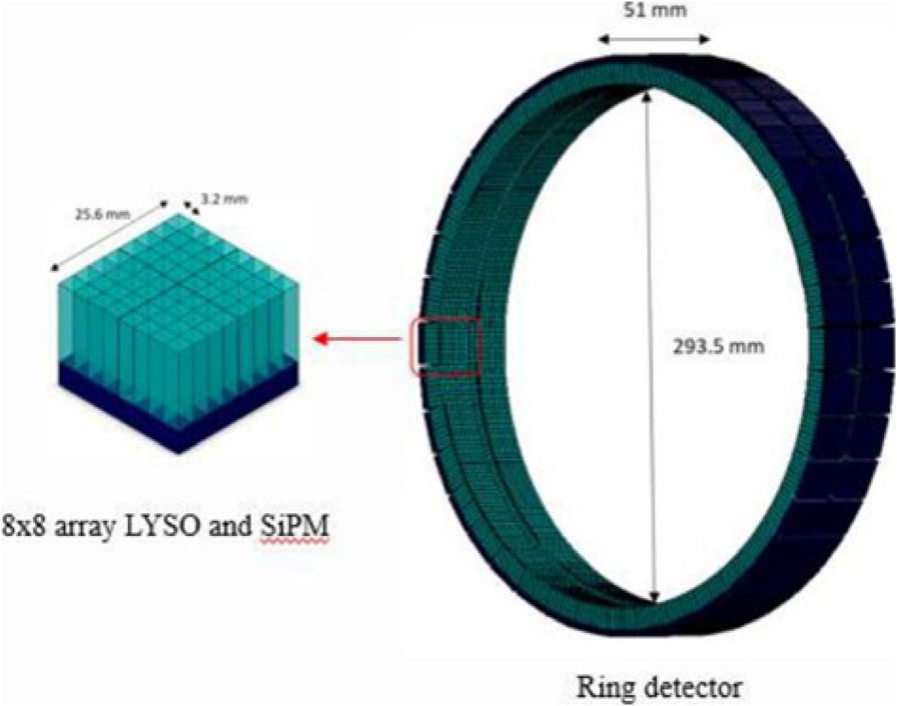
Geometry of brain PET for simulation

### A26 Total-body TOF-PET: 2 meters or 1.4 meters?

#### Weijie Tao^1,2^, Fenghua Weng^1^, Xiang Hong^1^, Gaoyu Chen^1^, Yunlong Zan^3^, Qiushi Ren^4^, Qiu Huang^1,2^

##### ^1^School of Biomedical Engineering, Shanghai Jiao Tong University, Shanghai, 200240, China; ^2^Department of Nuclear Medicine, Ruijin Hospital, Shanghai, 200240, China; ^3^University of Michigan - Shanghai Jiao Tong University Joint Institute, Shanghai Jiao Tong University, Shanghai, 200240, China; ^4^Department of Biomedical Engineering, College of Engineering, Peking University, 100871, Beijing, China

###### **Correspondence:** Qiushi Ren (qiuhuang@sjtu.edu.cn); Qiu Huang (renqsh@coe.pku.edu.cn)

**Background**

Total-body PET systems with larger AFOV have two essential advantages: (1) a higher geometric sensitivity, (2) a unique ability to scan multiple organs and body regions. The EXPLORER scanner is a 2-meter PET system [1,2] that covers the entire body of an adult. However, majority of the important organs are in a region ~1 meter in length. There is no need to include the lower limbs when imaging the entire body simultaneously. We have conducted simulation-based design study to compare the performance of a 1.4-meter system with the 2-meter system using the GATE toolkit.

**Methods**

The simulated 1.4 m long PET system consists of 26 axial block rings with an axial gap of 3.42 mm between adjacent rings. Each ring has 48 51×51 mm^2^ detector modules forming a ring of 800 mm in diameter. Each detector module consists of an array of 15×15 LYSO crystals with a crystal pitch of 3.42 mm. The 2-meter system was also simulated for comparison.

A 1.8-m line source was inserted in the center of a cylindrical water phantom (0.3 m in diameter, 1.8 m in length) to assess the sensitivity. A water cylinder phantom (100 cm long and 20 cm in diameter) with a line source was utilized for the NECR test.

**Results**

Figure 1 shows the sensitivity profiles. The total sensitivity of the 1.4-meter system is 69.72% of the 2-meter system. The sensitivity of 1.4-meter system is 93.45% of the 2-meter system in the 1.0 meter AFOV. The NECR curves of the scanners are shown in Fig. 2. The peak-NECR of the 2-meter system is 12.4% higher.

**Discussion**

There is no significant difference between the sensitivity of the 1.4-meter and 2-meter system when imaging a 1-meter region. Considering the complexity and cost of the 2-meter system, we would suggest the 1.4-meter system with 150 ps TOF [3] and 3 mm DOI [4] capabilities. The 1.4-meter system has a lower multiple coincidence event rate than the 2-meter system. A high TOF resolution will not only improve the effective sensitivity of the system, but also effectively reduce the possibility of the multiple coincidence events. We are currently assessing the effects of the 3 mm FWHM DOI on spatial resolutions and the effects of 150 ps FWHM CTR on the effective sensitivity and image quality.

**References**

1. Poon J K. The Performance Limits of Long Axial Field of View PET Scanners. Dissertations and Theses – Gradworks. 2013.

2. Isnaini I, Obi T, Yoshida E, et al. Monte Carlo simulation of sensitivity and NECR of an entire-body PET scanner. Radiological Physics and Technology. 2014; 7(2):1-8.

3. Q. Peng, W. S. Choong, C. Vu, et al. Performance of the Tachyon Time-of-Flight PET Camera. IEEE Transactions on Nuclear Science. 2015. 62(1): 111-119.

4. Blinder S, Camborde M L, Buckley K R, et al. Influence of depth of interaction on spatial resolution and image quality for the HRRT. Nuclear Science Symposium Conference Record. IEEE, 2005:5 pp.

**Fig. 1 (abstract A26).**
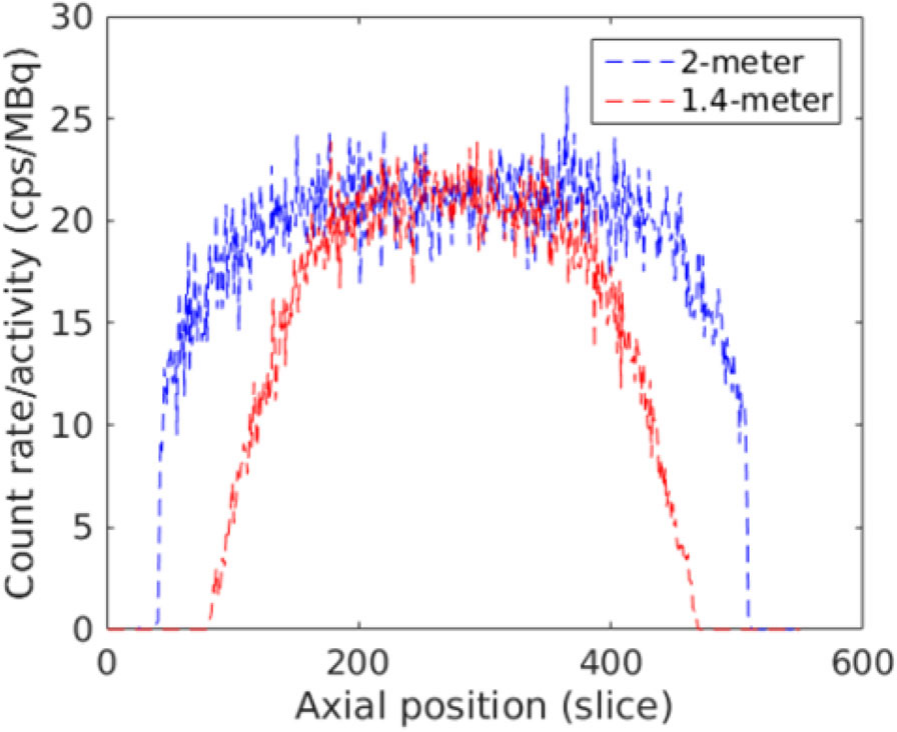
Sensitivity profiles

**Fig. 2 (abstract A26).**
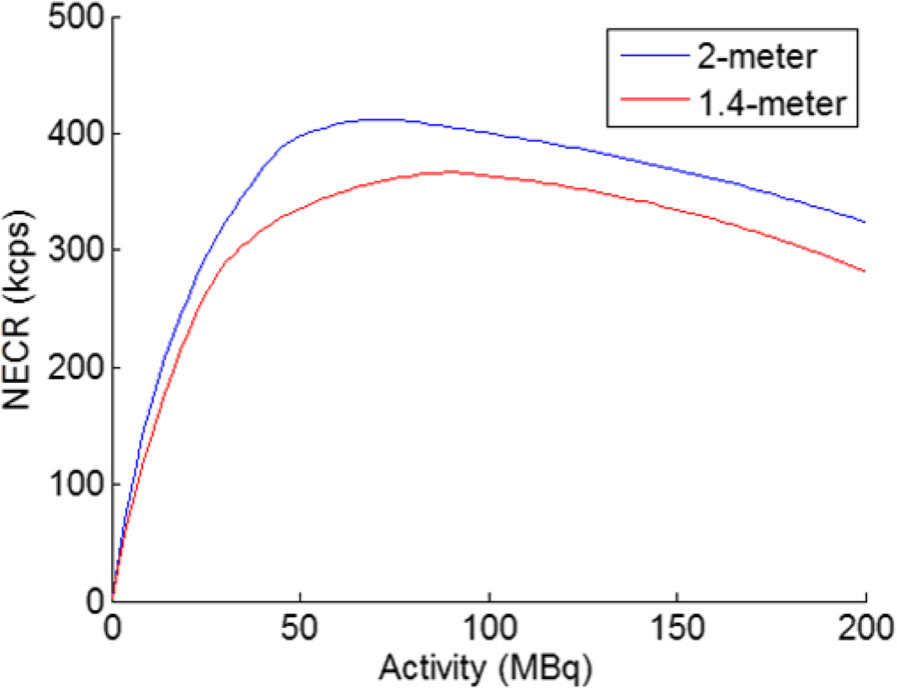
NECR curves

### A27 Hybrid ^18^F-FDG kinetic model for bias propagation reduction in Total-Body PET direct parametric image reconstruction

#### Thibaut Merlin, Dimitris Visvikis

##### INSERM, UMR1101, LaTIM, Universite de Bretagne Occidentale, Brest, France

###### **Correspondence**: Thibaut Merlin

**Introduction**

Direct dynamic PET image reconstruction allows to extract parametric images with higher signal to noise ratio. While this methodology has been mostly used in brain imaging, several works explored its potential interest for whole-body PET imaging [1]. The field of view (FOV) of standard PET systems requires specific care on the imaging protocol in order to acquire relevant dynamic frames for specific regions of interest. This burden can be potentially alleviated by Total-Body PET systems with extended FOVs, favoring the use of short-lived radiotracers and/or more complex kinetic models for whole-body dynamic exploration. On the other hand, parametric images estimated with direct parametric reconstruction algorithms can suffer from bias propagation in body regions which do not accurately follow a given kinetic model [2]. This aspect would cause potentially more complications for Total-Body image dynamic reconstruction as regions located in the extended FOV will unavoidably feature larger variety kinetic behaviors. Several works have tackled this shortcoming by using hybrid model approaches, such as involving a secondary dynamic model to fit residuals generated from difference between the data and a primary perfusion kinetic model [3].

**Material and Methods**

The present study extends the work in [3] to the estimation of FDG micro-parameters in a total-body acquisition set-up with the use of data-driven linear temporal basis functions [4] as the secondary model. As in the original work, generalised cross validation is used to weigh the estimate of the secondary model and penalize them in the voxels where the primary model correctly fits the data. The proposed reconstruction algorithm follows a standard 2-steps alternative estimation scheme, where MLEM image reconstruction is performed during the first step, followed by kinetic modelling within the same iteration.

The proposed method was evaluated using analytical projections of the anthropomorphic XCAT phantom with a mMR-based system extended to 70cm axial FOV. A dynamic dataset was generated with different time-activity curves constructed from a standard 5 parameters (K1,k2,k3,k4,Bv) ^18^F-FDG kinetic model for most phantom organs, as well as dual input and modified models adapted to the kinetics of the liver and kidney regions respectively.

**Results and Future work**

Results show that the modelling scheme is able to improve the fit of the kinetics in the simulated dataset and reduce the propagation-related bias. Current work focuses on the evaluation of the method on 4D and 5D (including the simulation of respiratory motion) Monte-Carlo simulated data, and the comparison of the proposed secondary model to previously proposed models.

References

[1] Karakatsanis NA and al. Phys. Med. Biol., vol. 61:5456, 2016.

[2] Kotasidis FA and al. IEEE NSS/MIC, pp 2868 - 2874, 2011.

[3] Kotasidis FA and al. Phys Med Biol, 59(14):6061-6084, Aug 2014.

[4] Reader and al. Phys Med Biol, 51(21):5455-5474, Nov 2006.

### A28 Preliminary investigation of ATCA electronics for total body PET data processing

#### Yingjie Wang^1,2^, Chien-Min Kao^2^, Tiehui T. Liu^3^, Jamieson Olsen^3^, Long Wei^1^, Chin-Tu Chen^2^

##### ^1^Beijing Engineering Research Center of Radiographic Techniques and Equipment, Institute of High Energy Physics, Chinese Academy of Sciences, Beijing, 100049, China; ^2^Department of Radiology, The University of Chicago, Chicago, Illinois, 60637, USA; ^3^Fermi National Accelerator Laboratory, Batavia, Illinois, 60510, USA

###### **Correspondence:** Chin-Tu Chen (c-chen@uchicago.edu)

A total-body (TB) PET system, due to its exceptional sensitivity, can produce events at extraordinarily high rates and efficient processing of these events can pose a major challenge. In this work, we investigate using the Pulsar board for on-the-fly event processing and coincidence filtering (CF) of such systems. Pulsar (Fig. 1) is a powerful and versatile electronics board developed at the Fermi Lab to provide rich-feature analysis of the massive amount of data generated during the short duration of a collision event. Based on the Advanced Telecommunications Computing Architecture (ATCA), it has a scalable architecture and is abundant in offering flexible, non-blocking, and high bandwidth. A single Pulsar II board can support 1Tbps and its flexible architecture allows multiple boards to communicate within one ATCA crate; therefore, a multiple-Tbps system can be easily configured within one crate hosting up to 12 Pulsars and we have built a test stand that demonstrated 4.8Tbps throughput (Fig. 2). The IO capability of the most recent board, Pulsar3a (Fig. 3), has increased to ~2Tbps.

Pulsar is also equipped with high-end field programmable gate arrays (FPGAs) for providing fast and versatile processing. We implement Content-Addressable Memory (CAM) using these FPGAs to parallelize and greatly accelerate CF and other event processing. For testing, we simulated a TB PET system using Geant4. The system, having an inner diameter of 79 cm and an axial length of 200 cm, contains 290,304 LYSOs of 3.9x3.9x15 mm^3^ in size. A cylinder phantom of 30-cm diameter and 180-cm length, filled with 2mCi F-18, was placed at the center of the system. For each simulated single event, the crystal index, time, and deposited energy were saved in list mode. The resulting simulation showed a data rate of 4Gbps. Using our test stand, which is capable of 4.8Tbps, we use the lower ACTA shelf to feed the simulation data according to the time stamps, thereby emulating realistic data stream from a TB PET system, to the upper ACTA shelf where event processing is implemented.

The data will be processed to identify coincidences using two methods: (1) the conventional sequential search method implemented on PC, and (2) the proposed CAM-based parallel search method implemented on Pulsar. The results will be compared to validate the CAM-based method. The saving in the CF processing time by Pulsar over PC will be assessed and we will evaluate whether the former can support on-the-fly CF for TB PET systems.

**Fig. 1 (abstract A28).**
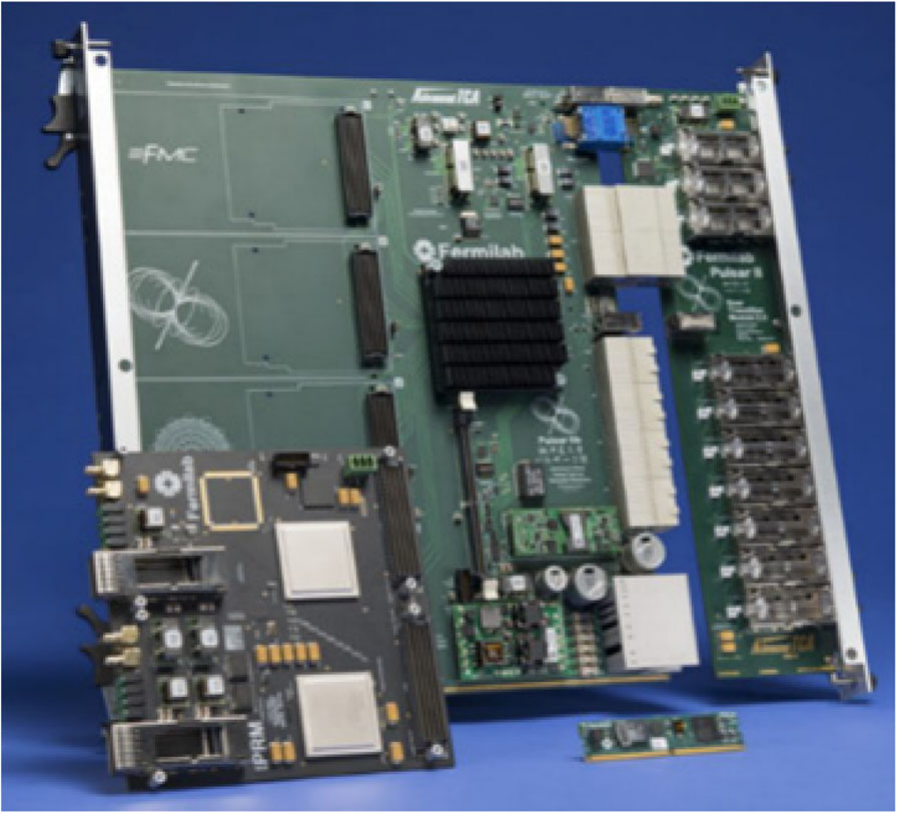
The Pulsar II board

**Fig. 2 (abstract A28).**
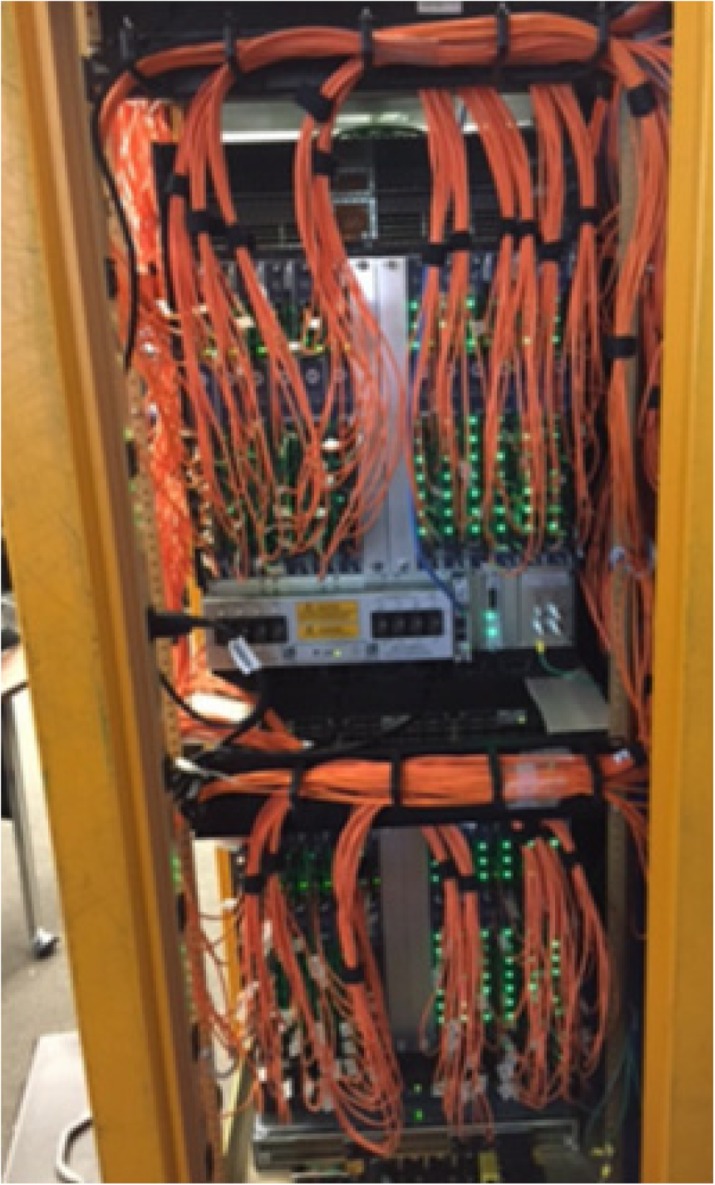
The Pulsar II test stand containing a lower ACTA shelf configured to emulate realistic data streams from a real detection system and an upper ACTA shelf for processing/pattern recognition

**Fig. 3 (abstract A28).**
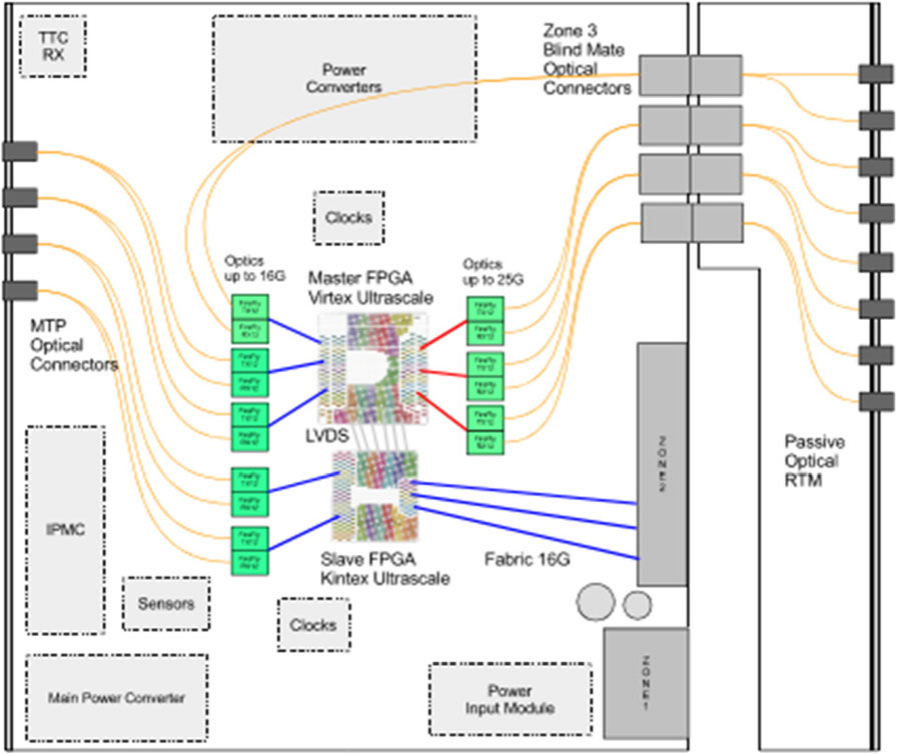
The Pulsar3a block diagram

### A29 NEMA NU 2012 Performance Evaluation of Silicon-Photomultiplier–Based and conventional PMT-based Time-of-Flight systems

#### Paulo Caribé^1,5^, Michel Koole^3^, Hugo Bertin^4^, Yves Dasseler^2^, Stefaan Vandenberghe^1^

##### ^1^Medical Imaging and Signal Processing, MEDISIP, Gent, Belgium; ^2^Department of Nuclear Medicine, UZ/Ghent, Gent, Belgium; ^3^Division of Nuclear Medicine, UZ/KU, Leuven, Leuven, Belgium; ^4^General Electric Healthcare, BeNeLux, Diegem, Belgium; ^5^National Council for Scientific and Technological Development, CN Pq, Sao Paulo, Brasil

###### **Correspondence:** Paulo Caribé (Paulo.Caribe@UGent.be)

The aim of this study is to assess the performance characteristic of new-generation SiPM-based and conventional PMT-based time-of-flight PET systems. For this purpose, NEMA NU 2–2012 performance measurements for characterizing spatial resolution (SR), sensitivity, image quality (IQ), noise equivalent count rate (NECR) and linearity were performed on GE Signa integrated PET/MR Discovery MI PET/CT (DMI) and Biograph mCT Flow PET/CT (Biograph).

**METHODS:**

NEMA NU-2 2012 testing was performed independently on GE Signa integrated PET/MR, installed at Katholieke Universiteit Leuven and the others at Ghent University. For the SR measurements, a ^18^F-FDG point source inside a glass capillary tube was positioned at 1 and 10 cm off-center in the field of view. Sensitivity tests at both institutions, plastic tubing (70 cm in length, 1 mm in inner diameter) was filled with an averaged calibrated activity of approximately 20.0 MBq of ^18^F-FDG The line source was placed in an aluminum sleeve ensuring complete annihilation of all positrons. NECR were measured using a 70-cm-long polyethylene cylinder with a diameter of 20 cm and a line source inserted axially into the cylinder 4.5 cm off-centered filed with 871.0, 883.0 and 865.0 MBq of ^18^F-FDG (at first frame start) for all systems. PET image quality tests were evaluated using the NEMA IQ phantom with a 4:1 ratio for the hot sphere to background activity concentration, which equals 52.0 MBq for a 9800 ml phantom. The scatter phantom line source was filled with activity between 116 – 120 MBq at scan start. For all systems, the accuracy of the attenuation and scatter correction were determined from the uniform background and could lung insert regions.

**RESULTS:**

The contrast recovery for small spheres is better for the Discovery MI 4 rings than for any of the other commercially available systems in Table 1. This better contrast recovery should lead to an improvement in the system’s ability to detect, visualize, and quantify smaller lesions.

Table 2 summarizes important counting rate metrics measured at both UGhent and KU Leuven.

The spatial resolution testing showed that, taken as a whole over all 3 resolution directions and the different distances from the center of the FOV, the Discovery MI performs comparably to the other systems in Table 1. The sensitivity of the Discovery MI is the highest of all the PET/CT systems although still lower than that of the GE Signa PET/MR system, with longer PET axial FOVs and smaller transaxial FOVs.

**CONCLUSION:**

NEMA NU-2 2012 testing of the SiPM-based Discovery PET/CT systems points to improved diagnostic sensitivity for small lesions and a wide range of promising applications, from low-dose oncology studies to high-dose studies with short-lived isotopes. However, sensitivity and counting rate measurements were substantial different as compared to GE Signa PET/MR system, with longer PET axial FOVs and smaller transaxial FOVs. In addition, comparisons with other PET/CT systems demonstrate the substantial performance improvements possible with the new generation of SiPM-based TOF PET/CT systems.

**Table 1 (abstract A29).** Shows the image quality, spatial resolution and sensitivity results for both institutions

Parameter	GE Healthcare			Siemens Healthcare
	Discovery MI PET/CT 3 Rings	Discovery MI PET/CT 4 Rings	SIGNA PET/MR	Biograph mCT Flow PET/CT
Contrast Recovery [%]				
10 mm–Radioactive	44.2	53.7	48.7	28.5
13 mm–Radioactive	53.4	64.0	62.9	42.3
17 mm–Radioactive	66.4	73.1	68.1	58.4
22 mm–Radioactive	70.7	82.7	76.1	70.7
28 mm–Non-Radioactive	81.6	86.8	87.1	72.1
37 mm–Non-Radioactive	84.5	90.7	92.7	78.3
Lung Error [%]	7.8	4.4	1.6	5.6
Spatial Resolution FWHM*				
Radial, 1 cm	4.65	4.10	4.46	4.33
Axial, 1 cm	4.47	4.48	5.35	4.25
Tangential, 1 cm	4.36	4.19	4.08	4.33
Radial, 10 cm	5.54	5.47	5.81	5.16
Axial, 10 cm	5.44	6.01	6.75	5.85
Tangential, 10 cm	4.75	4.49	4.44	4.72
Radial, 20 cm	7.41	7.53	8.42	5.55
Axial, 20 cm	5.78	6.10	7.30	7.80
Tangential, 20 cm	5.18	4.90	5.27	6.48
Sensitivity – Center of FOV				
(cps/kBq)	7.26	13.7	22.9	9.60

*Filtered backprojection

**Table 2 (abstract A29).** Counting Rate Measurements

Type of measurement *	GE Healthcare			Siemens Healthcare
	Discovery MI PET/CT 3 Rings	Discovery MI PET/CT 4 Rings	SIGNA PET/MR	Biograph mCT Flow PET/CT
Scatter Fraction at Peak NECR	41.7 %	40.4 %	43.4 %	33.5 %
Peak NECR	102.7 kcps	201.1 kcps	216.8 kcps	185 kcps
Activity at Peak NECR	24.70 kBq/ml	22.1 kBq/ml	18.6 kBq/ml	29.0 kBq/ml
Maximum Absolute Error	3.19 %	3.86 %	2.92 %	3.7 %

* ^18^F-FDG PET imaging

### A30 Potential role of whole body PET imaging to detect and characterize cardiovascular disorders

#### Koosha Paydary^1^, Sahra Emamzadehfard^1^, Saeid Gholami^1^, Sara Pourhassan^1^, Thomas J. Werner^1^, Poul Fleming Høilund-Carlsen^2^, Abass Alavi^1^

##### ^1^Department of Radiology, University of Pennsylvania, Philadelphia, PA, USA; ^2^Department of Nuclear Medicine, Odense University Hospital, Odense, Denmark

###### **Correspondence:** Abass Alavi (abass.alavi@uphs.upenn.edu)

**Background**

The purpose of this scientific communication is to discuss the potential impact of whole body PET instruments for assessing cardiovascular disorders with emphasis on global quantification of atherosclerotic burden.

**Materials and Methods**

The following preliminary data will be presented to demonstrate the necessity of such instruments in this setting. This research study enrolled 124 subjects which included 80 healthy volunteers and 44 patients with high risk for atherosclerosis who underwent NaF-PET/CT imaging. We quantified global molecular calcification in the thoracic aorta by assigning regions of interest throughout this artery. This assessment included ascending aorta (AA), aortic arch (AR) and descending aorta (DA). Thereafter, the average SUVmax, average SUVmean and global calcification score (GCS) were calculated for each segment and the entire aorta as a whole. We determined the degree of NaF uptake between healthy subjects and patients with risk factors. Furthermore, correlations between age and average SUVmax, average SUVmean and GCS were determined in both groups. Finally, multivariate linear regression and logistic regression models were explored to determine the predictability of the Framingham Risk Score (FRS) and of an unfavorable cardiovascular disease (CVD) risk profile by our measures of NaF uptake, respectively.

**Results**

Average SUVmax, average SUVmean and GCS were significantly higher in patient compared to healthy subjects (Table 1). Average SUVmax, average SUVmean and GCS in all vascular segments and whole vessel were correlated with age, although the correlation coefficients were higher in patients with high risk for atherosclerosis (Table 2). The correlation of average SUVmean with age was borderline significant in the healthy subjects (r= 0.32, P=0.04), while it was significantly higher in patients group (r= 0.64, P<0.001). GCS of the entire thoracic aorta (GCS total) was a stronger predictor of FRS as compared to average SUVmax and average SUVmean (Adjusted R^2^ =0.38, standardized β= 0.58, P-value <0.001). Also, GCS total was borderline significant predictor of unfavorable CVD risk profile as compared to average SUVmax and average SUVmean (Odds Ratio=1.006, 95% CI=1.000-1.013, P-value=0.05).

**Conclusions**

Active global calcification of thoracic aorta is correlated with age, and the extent of such correlation is higher among subjects with CVD risk factors such as hypertension and hypercholesterolemia. Therefore, global assessment of molecular calcification can predict unfavorable CVD risk profile. We believe instruments with large field of view will play a major role in whole body assessment of atherosclerosis and possibly other cardiovascular disorders.

**Table 1 (abstract A30).** The average SUVmax, average SUVmean and GCS of each vascular segment of thoracic aorta in our total sample including 124 subjects, and the two subgroups of healthy subjects and patients with high risk of atherosclerosis. The P-value column represents the significance of the differences between the healthy and patients groups (SUV: standardized uptake value, AR: aortic arch, AA: ascending aorta, DA: descending aorta, GCS: Global Calcification Score)

	Vascular segment	Total sample (n=124)	Healthy subjects (n=80)	Patients with high risk (n=44)	P value^*^
Average SUVmax (mean±SD)	AR	1.97±0.80	1.84±0.72	2.22±0.89	0.01
	AA	1.88±0.73	1.73±0.71	2.16±0.70	0.002
	DA	1.93±0.61	1.81±0.55	2.14±0.65	0.004
	total	1.93±0.61	1.79±0.56	2.15±0.64	0.002
Average SUVmean (mean±SD)	AR	0.94±0.34	0.87±0.30	1.07±0.37	0.002
	AA	0.90±0.25	0.83±0.20	1.02±0.29	<0.001
	DA	1.00±0.37	0.94±0.37	1.09±0.34	0.02
	total	0.94±0.28	0.88±0.23	1.06±0.32	0.002
GCS (mean±SD)	AR	36.17±20±53	31.85±17.85	44.03±22.84	0.001
	AA	67.90±36.6	56.56±29.91	88.50±39.02	<0.001
	DA	67.34±41.2	58.14±30.29	84.08±52.32	0.001
	total	171.42±87.96	146.56±69.24	216.62±100.43	<0.001

*independent sample t test

**Table 2 (abstract A30).** Correlations between measures of NaF uptake (Average SUVmean, Average SUVmax and GCS) in the three thoracic aorta segments as well as the whole vessel with increasing age and 10 year-cardiovascular disease risk using Framingham Risk Score (FRS) are shown. The correlations were evaluated among our total sample, patients group and healthy subjects separately. Numbers are representative of Pearson Correlation Coefficients (r) and their level of significance (P-values) in parentheses (AR: Aortic Arch; AA: Ascending Aorta; DA: Descending Aorta; SVC: Superior Vena Cava; GCS: Global Calcification Score; FRS: Framingham Risk Score; NS: Not Significant)

		Total sample (n=124)		Patients (n=44)		Healthy Subjects (n=80)	
		Age	FRS	Age	FRS	Age	FRS
Average SUVmean	AR	0.40 (<0.001)	0.30 (<0.001)	0.60 (<0.001)	0.39 (<0.001)	0.19 (NS)	0.05 (NS)
	AA	0.53 (<0.001)	0.38 (<0.001)	0.66 (<0.001)	0.43 (<0.001)	0.36 (0.001)	0.13 (NS)
	DA	0.28 (<0.001)	0.23 (0.01)	0.62 (<0.001)	0.39 (0.01)	0.06 (NS)	0.02 (NS)
	Total	0.43 (<0.001)	0.33 (<0.001)	0.64 (<0.001)	0.42 (<0.001)	0.20 (NS)	0.07 (NS)
Average SUVmax	AR	0.29 (<0.001)	0.29 (<0.001)	0.33 (<0.001)	0.36 (0.01)	0.18 (NS)	0.11 (NS)
	AA	0.35 (<0.001)	0.36 (<0.001)	0.43 (<0.001)	0.39 (0.01)	0.21 (NS)	0.21 (0.05)
	DA	0.33 (<0.001)	0.32 (<0.001)	0.53 (<0.001)	0.42 (<0.001)	0.13 (NS)	0.11 (NS)
	Total	0.35 (<0.001)	0.36 (<0.001)	0.51 (<0.001)	0.44 (<0.001)	0.16 (NS)	0.15 (NS)
GCS	AR	0.55 (<0.001)	0.53 (<0.001)	0.60 (<0.001)	0.49 (<0.001)	0.46 (<0.001)	0.46 (<0.001)
	AA	0.64 (<0.001)	0.57 (<0.001)	0.67 (<0.001)	0.41 (<0.001)	0.54 (<0.001)	0.56 (<0.001)
	DA	0.53 (<0.001)	0.48 (<0.001)	0.58 (<0.001)	0.38 (0.01)	0.44 (<0.001)	0.47 (<0.001)
	SVC	0.20 (0.02)	0.22 (0.01)	0.38 (0.01)	0.22 (NS)	0.07 (NS)	0.17 (NS)
	Total	0.65 (<0.001)	0.59 (<0.001)	0.70 (<0.001)	0.47 (<0.001)	0.55 (<0.001)	0.57 (<0.001)

### A31 A Gate Simulation study to compare small animal total body PET scan base on monolithic and pixelated detectors

#### Ahad Zeinali^1^, Meysam Dadgar^2^

##### ^1^Medical physics group leader, Medical Physics Department, Urmia medical science university, Urmia, Iran; ^2^Medical Physics Department, Urmia medical science university, Urmia, Iran

**Introduction**

One of the reasons which total body Positron Emission Tomography (PET) attracts attention to itself is the importance of every single event which carrying important information from the tissue by itself. Total body PET scans can accumulate more information by providing a large area of detection. Spatial resolution is one of the major factors in PET scan image qualities which can improve with decreasing crystal size, but dead space between crystals cause to decrease sensitivity. Another problem which shows itself in smaller scintillation crystals is parallax error which decreases resolution. In this study, we simulate small animals total body PET scan by Geant4 Application for Tomographic Emission, GATE. We evaluate the count rate in two different pixilated and monolithic scintillation detectors by different BGO, GSO, LSO and LYSO crystals.

**Materials and methods**

In this simulation, we use GATE. Both of the PET scans simulate in cylindrical geometry. Pixilated detectors PET scan simulate by 1×1×16 mm dimensions scintillation crystals. These crystals located in separated 32×24×200 mm blocks. At PET scan based on monolithic scintillation detectors, dimensions of the crystals are 20×20×16.

All of the simulations done in the 180 s. These simulations were done for BGO, GSO, LSO and LYSO scintillation crystals. In all of these simulations, there is a cylinder shape source, to evaluate the effect of source position we change its location after each simulation respectively 0, 5, 10, 15 and 20 mm from the center of the scanner.

**Results**

We simulated monolithic PET scan and set scan time in 180 s. After every simulation changed source position at intervals 5 mm from the center and do all these steps for BGO, GSO, LSO and LYSO scintillation crystals. As you can see in Fig. 1, it shows that by changing the source position count rate is increasing, but not so much in comparing by total count rate. Results also show that the count rate for BGO is highest and for LYSO is less than the other crystals.

In a pixilated crystal PET scan as you can see in Fig. 2, count rate increase, obviously for all of the crystals, but like monolithic detectors by a moving source from center to outside, we can see a smooth increase in count rate. With this scanner also highest count rate is for BGO and lowest is for LYSO.

**Conclusion**

In this simulation study, we only studied count rate of two pixilated arrays and monolithic detectors of PET scanner, results show that count rate for pixilated mode is higher than monolithic, we also checked the energy resolution and for all the simulations variances of energy resolution was incurable, but to achieve the best results we know that count rate is not the only parameter, there are many parameters which helps to get to the best results. For pixilated detectors, parallax error is major especially when crystal dimensions are very small. So for best results, we recommend considering all the parameters like count rate, spatial resolution, signal to noise ratio etc.

**Fig. 1 (abstract A31).**
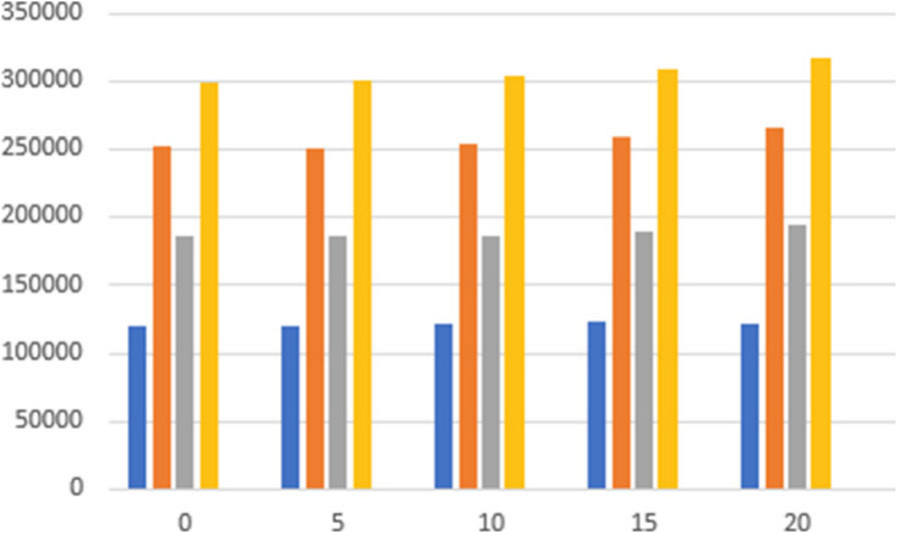
Count rate for BGO, GSO, LSO and LYSO crystals in monolithic detectors

**Fig. 2 (abstract A31).**
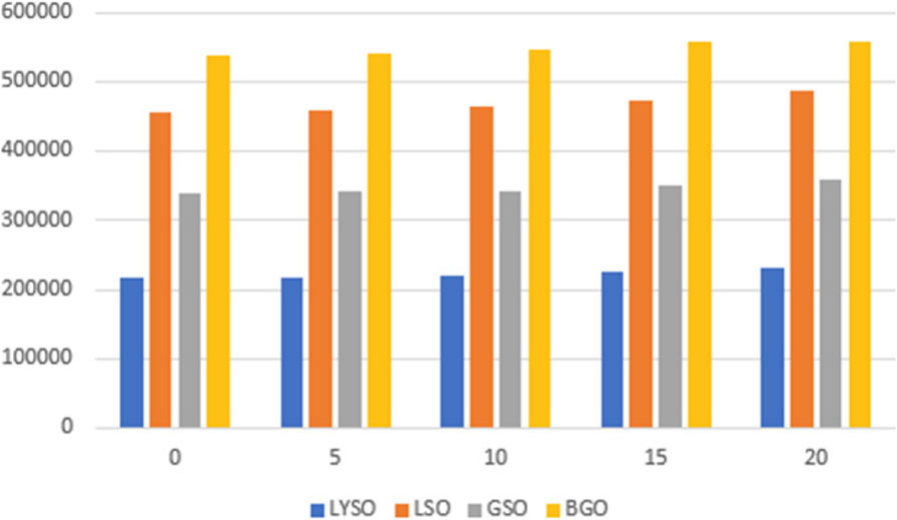
Count rate for BGO, GSO, LSO and LYSO crystals in pixelated detectors

### A32 Challenges and methodology of quantitative image reconstruction for the total-body PET

#### Xuezhu Zhang, Ramsey D. Badawi, Simon R. Cherry, Jinyi Qi

##### University of California, Davis, CA, USA

**Background**

The EXPLORER consortium is building the world’s first 2-meter long total-body PET scanner [1]. In this work, we describe the challenges and methodology of quantitative image reconstruction for the total-body scanner. We also evaluate the performance of the reconstruction method using two smaller-scale prototypes: the mini-EXPLORER-I (mini-I) and mini-EXPLORER-II (mini-II), which were built before constructing the 2-meter scanner [2].

**Materials and methods**

The EXPLORER total-body system consists of 564480 detector crystals, forming over 100 billion lines of response (LORs). When including time-of-flight TOF information, the number of all possible sinogram bins is over 1 trillion. A pre-computed system matrix after compression by geometric symmetries requires ~800 GB of storage space for a field of view of 69 cm with 2.85-mm cubic voxels (equal to the crystal size). This presents a daunting challenge for image reconstruction. For efficient computation, we have developed a list-mode TOF reconstruction method [3-4]. We performed demonstration phantom and animal studies on mini-I and mini-II. A system matrix with a multiple ray-tracing projector was calculated to model the detector response function, including geometric solid angle and photon penetration effects. Image reconstruction was regularized through the kernel method. We performed a component-based normalization using a scan of an annular source. Attenuation correction factors were obtained from a CT scan. Randoms were estimated from singles rates and verified by the delayed window method.

**Results**

Figure 1 shows reconstructed images of a 1-s frame from a 1-hour scan of a non-human primate using mini-I. The injected activity was 92.5 MBq. Compared with the OSEM reconstruction, the kernel based reconstruction delineates the tracer distribution more clearly. The kernel matrix was constructed using three composite images reconstructed from the 1-hour scan. Figure 2 shows a transaxial slice of the reconstructed images of a Derenzo phantom acquired on the mini-II scanner. The 1.6-mm hot rods can be resolved using our system matrix model.

**Conclusions**

We have implemented a list-mode TOF reconstruction method for the mini-EXPLORER scanners and demonstrated that noise properties and resolution can be improved through the use of appropriate regularization and resolution modeling. This approach holds promise for efficient implementation of quantitative image reconstruction on the total-body EXPLORER scanner.

**Acknowledgements**

Support for this work includes NIH grants R01 CA206187 and R01 CA170874. We would like to thank Drs. Martin S. Judenhofer and Eric Berg for providing the animal and phantom data.

**References**

1. Cherry SR, Jones T, Karp JS, Qi J, Moses WW, Badawi RD. Total-body PET: maximizing sensitivity to create new opportunities for clinical research and patient care. J Nucl Med. 2018; 59:3-12.

2. Badawi RD, Liu W, Berg E, Lv Y, Xu T, An S, Dong Y, Zhang X, Judenhofer MS, Qi J, Jones T, Tarantal AF, Bao J, Li H, Cherry SR. Progress on the EXPLORER project: towards a total body PET scanner for human imaging. SNMMI. 2018.

3. Zhang X, Zhou J, Cherry SR, Badawi RD, Qi J. Quantitative image reconstruction for total-body PET imaging using the 2-meter long EXPLORER scanner. Phys. Med. Biol. 2017; 62: 2465-2485.

4. Zhang X, Zhou J, Wang G, Poon J, Cherry SR, Badawi RD, Qi J. Feasibility study of micro-dose total-body dynamic PET imaging using the EXPLORER scanner. 2014; 55: 269.

**Fig. 1 (abstract A32).**
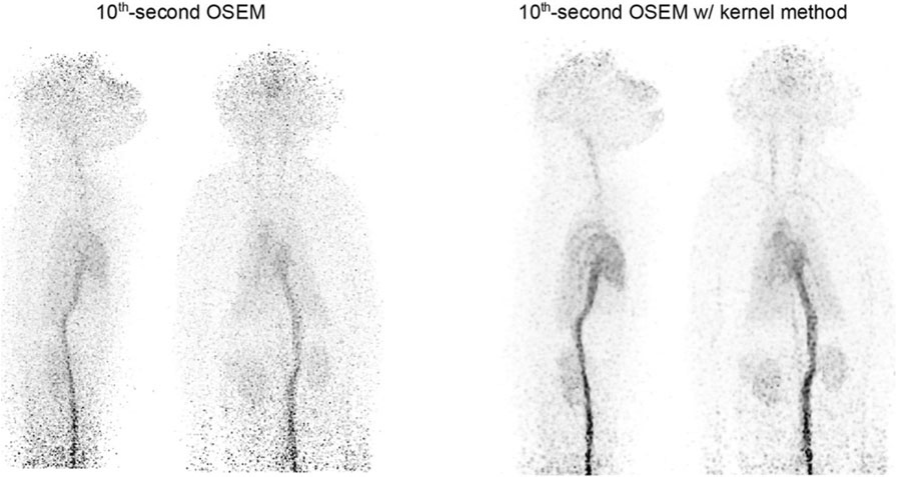
Reconstructed images of the primate ^18^F-FDG imaging study scanned using the mini-EXPLORER-I. OSEM (left) and kernel (right) reconstruction for the 1-second dynamic PET at the 10^th^ second post injection. The images are maximum intensity projections

**Fig. 2 (abstract A32).**
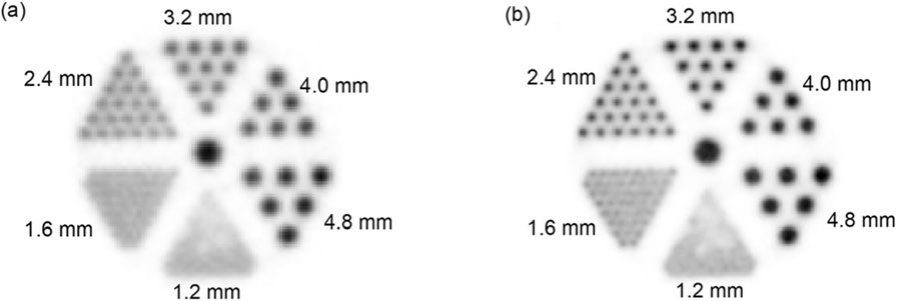
Fully-3D Image reconstruction of a Derenzo phantom scanned on the mini-EXPLORER-II without and with our resolution model. (a) Single-ray tracing projector w/o PSF modeling; (b) Pre-calculated system matrix w/multiple-ray tracing projector PSF modeling. The 1.6 mm hot rods can be resolved using our accurate system matrix modeling with the 2.85x2.85x18.1 mm^3^ crystal

